# Spin–Electric Coupling in a Cobalt(II)‐Based Spin Triangle Revealed by Electric‐Field‐Modulated Electron Spin Resonance Spectroscopy

**DOI:** 10.1002/anie.202017116

**Published:** 2021-03-09

**Authors:** Benjamin Kintzel, Maria Fittipaldi, Michael Böhme, Alberto Cini, Lorenzo Tesi, Axel Buchholz, Roberta Sessoli, Winfried Plass

**Affiliations:** ^1^ Institut für Anorganische und Analytische Chemie Friedrich-Schiller-Universität Jena Humboldtstrasse 8 07743 Jena Germany; ^2^ Department of Physics and Astronomy University of Florence and INSTM UdR via Sansone 1 Sesto Fiorentino (FI) Italy; ^3^ Dipartimento di Chimica “Ugo Schiff” Universitá degli Studi Firenze Via della Lastruccia 3–13 50019 Sesto Fiorentino (FI) Italy; ^4^ Current address: Institute of Physical Chemistry University of Stuttgart Pfaffenwaldring 55 70569 Stuttgart Germany

**Keywords:** cobalt, electron spin resonance spectroscopy, magnetic properties, spin–electric effect, spintronics

## Abstract

A cobalt(II)‐based spin triangle shows a significant spin–electric coupling. [Co_3_(pytag)(py)_6_Cl_3_]ClO_4_⋅3 py crystallizes in the acentric monoclinic space group P2_1_. The intra‐triangle antiferromagnetic interaction, of the order of ca. −15 cm^−1^ (**H**=−JS_a_S_b_), leads to spin frustration. The two expected energy‐degenerate ground doublets are, however, separated by a few wavenumbers, as a consequence of magnetic anisotropy and deviations from threefold symmetry. The Co_3_ planes of symmetry‐related molecules are almost parallel, allowing for the determination of the spin–electric properties of single crystals by EFM‐ESR spectroscopy. The spin–electric effect detected when the electric field is applied in the Co_3_ plane was revealed by a shift in the resonance field. It was quantified as Δg_E_/E=0.11×10^−9^ m V^−1^, which in terms of frequency corresponds to approximately 0.3 Hz m V^−1^. This value is comparable to what was determined for a Cu_3_ triangle despite the antiferromagnetic interaction being 20 times larger for the latter.

## Graphical abstract

This contribution describes the observation of spin–electric coupling utilizing electric‐field‐modulated ESR (EFM‐ESR) spectroscopy for single‐crystals of a spin triangle which is characterized by the strong magnetic anisotropy of the individual cobalt(II) centers and a pronounced sensitivity of the molecular energy levels on the relative orientation of their magnetic axes as well as the exchange coupling between them.

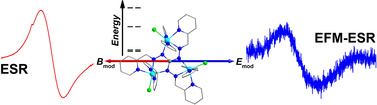

Quantum computation is a cutting edge subject because of its potential implications for information technology. Significant advances have been achieved in recent years,^[1]^ mainly thanks to the successful development of superconducting quantum bits (qubits). An attractive alternative to provide electronic quantum spins as qubits are molecular magnets,^[2]^ as they offer the advantage of chemically tunable properties.^[3]^ Magnetic fields are hardly suitable to control electron spins because of their poor spatial locality, whereas electric fields are a convenient alternative due to the fact that strong electric fields can be applied at high spatial resolution.^[4]^ The coupling between electron spins and an applied electric field is generally known as spin–electric coupling and has been observed in systems with appropriate spin–orbit interactions.^[5]^

At the molecular level, a spin–electric effect has been proposed for the special molecular arrangement of antiferromagnetically coupled non‐integer spins,[^6^, ^7^] a phenomenon known as spin frustration.[^8^, ^9^] The most straightforward realization of a spin‐frustrated system on the molecular level is an equilateral triangular arrangement of three non‐integer spins, as demonstrated by the numerous examples of such compounds fulfilling the above‐mentioned condition.^[10]^ As a specific feature of such spin‐frustrated systems, this leads to two quasi‐degenerate Kramers doublets as ground state, for which, due to the lack of inversion symmetry, in‐plane electric fields can couple states of opposite spin chirality. Moreover, Dzyalozhinsky–Moriya interactions due to spin–orbit interactions in the molecule, although not necessary for the observation of the spin–electric effect, are able to couple spin and chirality within the ground‐state multiplet.[^6^, ^7^] At this point it is worth noting that the spin–electric effect for the above‐mentioned spin systems, assuming a rigorous threefold symmetry, is predicted to be dominated by the modification of the Heisenberg exchange *J* within the molecule rather than by the modification of the Dzyalozhinsky–Moriya interaction *G*, with the relative influence being approximately proportional to their ratio (|δ*G*|/|δ*J*|≈|*G*|/|*J*|).^[7]^

Electric field effects on the electron spin resonance (ESR) spectra were initially employed to extract information on the symmetry of the coordination environment.^[11]^ Recently, different ESR‐spectroscopic setups have been designed to investigate the possible coupling of molecular spin systems to electric fields,[^12^, ^13^, ^14^] all of them featuring specific advantages and drawbacks. When the spin system shows a long coherence time, echo detection under the effect of an electric field pulse allows for the quantification of spin–electric coupling also in randomly oriented samples. In the case of cobalt(II), which usually exhibits broad linewidths and therefore short coherence times, continuous wave (CW)‐ESR spectroscopy with detection under electric field modulation (EFM)[^14^, ^15^] is better suited and apt to perform single‐crystal measurements. At difference from experiments where transitions are induced by the electric field of the microwave radiation, in the experiments discussed here, the electric field only affects the static Hamiltonian, leaving the selection rules unaltered.

In general, given the local character of the ESR transitions, linear electric field effects can be observed also in centrosymmetric crystals or frozen solutions,[^13^, ^16^, ^17^] provided the active site lacks the inversion center. However, to make use of the single‐crystal EFM‐ESR technique for a first‐order detection, the presence of a permanent electric dipole moment is required, which can be ensured by the lack of crystallographic inversion symmetry.[^11^, ^14^, ^15^] A compound fulfilling these prerequisites^[18]^ has been published by some of the authors and is based on octahedrally coordinated cobalt(II) ions as spin centers, which also provide the spin–orbit coupled nature of the cobalt(II) ion. However, this compound contains crystallographically equivalent molecules in two different orientations with nearly orthogonal Co_3_ planes, which basically hampers an experiment intending to address effects due to electric fields applied within the Co_3_ planes.

Herein, we report on the synthesis of the compound [Co_3_(pytag)(py)_6_Cl_3_]ClO_4_⋅3 py (**Co3P**), a trinuclear pseudo‐*C*_3_ symmetric cobalt(II) cationic complex based on the Schiff‐base ligand H_2_pytag (1,2,3‐tris[(pyridine‐2‐ylmethylidene)amino]guanidine; Supporting Information, Figure S1 and section Experimental Procedures), which possesses the chemically identical molecular structure as the previously reported modification [Co_3_(pytag)(py)_6_Cl_3_]ClO_4_⋅3.5 py (**Co3C**) crystallizing in the acentric space group *C*_2_ with a slight difference in solvent content.^[18]^ The new compound **Co3P** presented here is characterized by single‐crystal X‐ray analysis,^[19]^ magnetic susceptibility data, CW ESR experiments, and single‐crystal EFM‐ESR measurements. The experimental data is supplemented by computational studies based on broken‐symmetry DFT and ab initio CASSCF/CASPT2 calculations.

**Co3P** crystallizes in the monoclinic polar space group *P*2_1_ with one cationic trinuclear cobalt(II) complex molecule, one perchlorate anion, and three co‐crystallized pyridine molecules in the asymmetric unit (crystallographic and structural information is given in the Supporting Information, Table S1). The molecular structure of the complex cation [Co_3_(pytag)(py)_6_Cl_3_]^**+**^ as found in **Co3P** is depicted in Figure 1. All three cobalt(II) centers occupy one tridentate N_3_ pocket of the tritopic deprotonated pytag^2−^ ligand. Their coordination sphere is further saturated by two *trans* pyridine N donor atoms and one chlorido ligand within the plane defined by the N_3_ donor set of the tritopic chelate ligand resulting in a six‐coordinate N_5_Cl distorted octahedral coordination environment for all three cobalt(II) centers. A more detailed analysis of the coordination environment (Tables S2 and S3), the intermolecular interactions (Figure S2), as well as a comparison with the structure of the complex cation in **Co3C** (Figure S3) are given in the Supporting Information.

**Figure 1:**
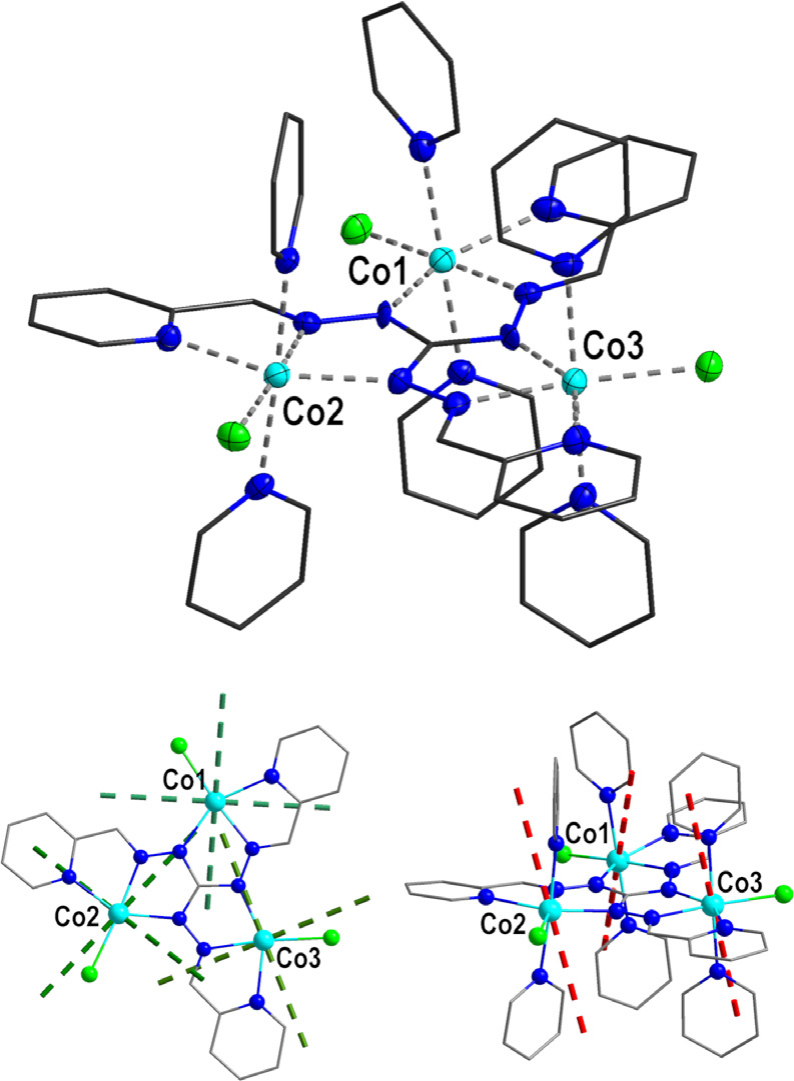
Top: molecular structure of the complex cation [Co_3_(pytag)(py)_6_Cl_3_]^+^ of **Co3P**. Donor and metal atoms are depicted at 50 % probability level. Hydrogen atoms are omitted for clarity. Bottom: Ab initio calculated (*S*_eff_=1/2 ) main anisotropy axes for the ground state Kramers doublet of the three individual cobalt(II) centers in **Co3P** (hydrogen atoms omitted for clarity). Bottom Left: the easy‐plane anisotropy given by the two easy axes at the cobalt centers (green dashed lines; pyridine ligands are not displayed). Bottom right: hard axes of magnetization of the cobalt centers (red dashed lines).

Due to crystal symmetry, a second cationic trinuclear complex unit of **Co3P** is generated by a twofold screw axis along the crystallographic $\vec{b}$ axis. This leads to a situation where the Co_3_ planes of both symmetry‐related complex cations are accidentally parallel to the $\vec{b}$ axis with a deviation of less than 0.3°, thus leading to a parallel orientation of the planes of both trinuclear molecules (Supporting Information, Figure S2). This feature is crucial for studying all molecules with the electric field applied in the plane of the triangle. Moreover, despite the structural similarity with the modification **Co3C** (Supporting Information, Figure S3 and Table S3), a magnetic and theoretical characterization of the new derivative **Co3P** is essential and cannot be based on the previously reported data of **Co3C**.^[18]^ In particular, the magnetic properties of octahedral cobalt(II) centers, besides to variations in bond lengths and angles, are very sensitive to seemingly subtle structural changes, such as the torsion angle *ϑ* of the aromatic planes of the two *trans* pyridine co‐ligands (for more details, see the Supporting Information, Figure S4 and Table S4).^[20]^

Temperature‐dependent magnetic measurements of a ground crystalline powder sample of **Co3P** from 2 to 300 K at an applied dc field of *H*_dc_=1000 Oe provided the *χ*_M_ *T* data visualized in Figure 2 (for the low‐temperature regime, see the Supporting Information, Figure S5). The value of 7.42 cm^3^ K mol^−1^ at 300 K is markedly higher than the expected spin‐only value for three independent high‐spin cobalt(II) centers with *S=*3/2 of 5.64 cm^3^ K mol^−1^ (for *g=*2), which is indicative for a significant orbital contribution as it is known for octahedrally coordinated cobalt(II) ions. Upon lowering the temperature, *χ*_M_ *T* decreases, which suggests an antiferromagnetic exchange. Below 50 K the slope in *χ*_M_ *T* is reduced, with an additional step below 5 K being observed. The non‐zero low temperature value of *χ*_M_ *T* is in accordance with the expected geometrically frustrated spin ground state.[^8^, ^9^]

**Figure 2:**
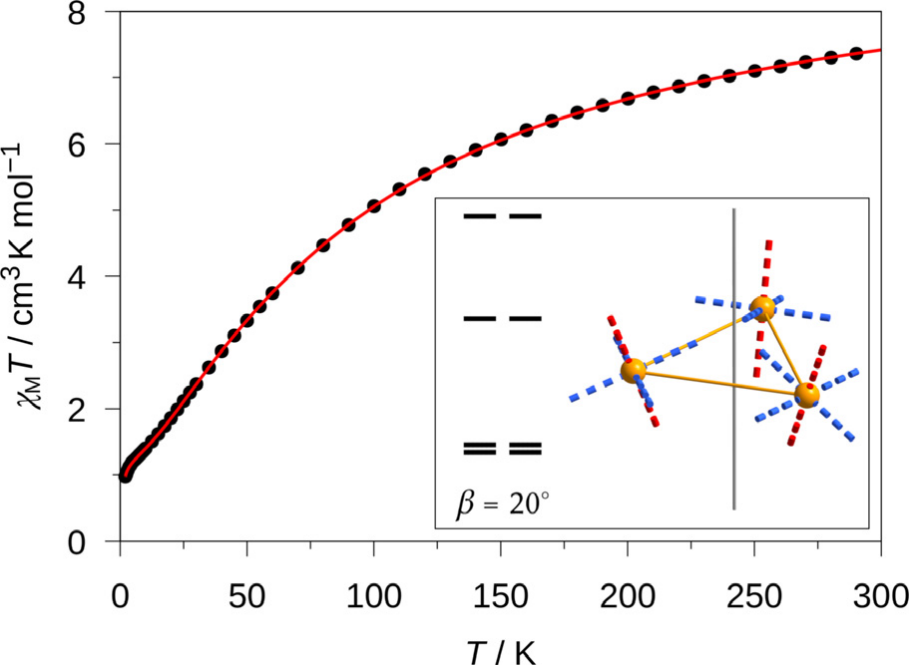
Temperature dependence of the magnetic susceptibility data *χ*_M_ *T* (•) of **Co3P** at an applied static magnetic field *H*_dc_ of 1000 Oe. The red solid line is the best‐fit curve according to the Hamiltonian given in Equation (1). Inset: the low energy state structure resulting from the simulation applying an intersecting angle *β*=20° (for further details see the Supporting Information, Figure S6).

A fit of the *χ*_M_ *T* data according to the Hamiltonian given in Equation 1 was performed utilizing a simplified, yet authentic representation of the effects involved. The Hamiltonian includes the magnetic exchange (**H**_ex_), spin–orbit interactions (**H**_SO_), ligand‐field splitting (**H**_LF_), and Zeeman interaction (**H**_Ze_). To avoid overparametrization, the number of fitting parameters was reduced by assuming a pseudo‐*C*_3_ symmetry in **Co3P** (see section Magnetic Susceptibility in the Supporting Information).

(1) $$H=H{}_{\mathrm{ex}}+H{}_{\mathrm{SO}}+H{}_{\mathrm{LF}}+H{}_{\mathrm{Ze}}$$

The obtained best fit is included in Figure 2 (see also Supporting Information, Figure S5) and the parameters are listed in the Supporting Information, Table S5. The exchange interaction parameter is determined to *J*_ex_=−14.3 cm^−1^, which is somewhat larger than the value reported for the corresponding **Co3C** (−12.6 cm^−1^).^[18]^ The derived parameters that describe the single‐ion magnetic anisotropy indicate an easy‐plane anisotropy for the individual cobalt(II) centers, which agrees with the results found for **Co3C**. However, since the local symmetry at each cobalt(II) center is very low, an additional Euler angle of rotation *β* was introduced, describing the angle of intersection between the local magnetic *z* axis (hard axis) and the pseudo threefold axis. This angle has a major effect on the energy pattern of the low‐lying spin states, as depicted in the Supporting Information, Figure S6, for four different *β* angles in the 0–90° range. With the best‐fit value *β=*20.6° a good agreement could also be obtained for the overall low‐temperature behavior of **Co3P** (see Supporting Information, Figure S5), which results in an experimentally determined energy difference between the two ground Kramers doublets (KDs) of *E*_KD2_−*E*_KD1_=1.9 cm^−1^.

This overall picture was confirmed with the help of computational approaches. First, magnetic exchange interactions have been evaluated by broken‐symmetry DFT (BS‐DFT) calculations (see the Supporting Information, section Computational Details and Figure S7). Three antiferromagnetic individual coupling constants (*J*_12_, *J*_13_, and *J*_23_) were evaluated for **Co_3_P** with values between −16.2 cm^−1^ and −18.8 cm^−1^ (Supporting Information, Table S6), thus confirming the validity of our assumption of pseudo‐*C*_3_ symmetry within the best‐fit procedure. The corresponding spin density plots for **Co3P** are visualized in the Supporting Information, Figure S8, showing that the magnetic exchange is mainly mediated by the N−N diazine moiety of the tritopic ligand.

Ab initio calculations for the three crystallographically independent cobalt(II) ions in **Co_3_P** were performed to obtain insight into their magnetic anisotropy (see Supporting Information, Figure S9 for computational models and section Computational Details). Relative CASSCF/CASPT2/SO‐RASSI energies (Supporting Information, Tables S7–S10) show a threefold ^4^T_1g_ [^4^F] ground multiplet for all three cobalt(II) centers as expected for octahedral high‐spin cobalt(II) ions. The energy gap between individual ground and excited KDs ranges from 97 to 205 cm^−1^. A comparison with the values determined by the fit of the experimental data, given in the Supporting Information, Figure S10, indicates a good agreement. The directions of the largest and smallest component of the *g*‐tensor for the ground doublet (*S*_eff_=1/2 ) are depicted in Figure 1 for the individual cobalt(II) centers. A slightly rhombic easy‐plane anisotropy is computed for each center in terms of *g* factors (*g*_*x*,*y*_>*g_z_*; see Supporting Information, Table S11, for the full list of parameters), which is in good agreement with an anticipated easy‐plane anisotropy as obtained by the best fit of the experimental data ($B_{2}^{0}$ >0; see Supporting Information, Table S5). Interestingly, the angles of intersection between the normal to the Co_3_ plane and the ground state hard axis for Co1/Co2/Co3 are 27.2°/20.4°/13.9°, respectively, which is in good agreement with the best‐fit *β* value derived from the experimental data. For the first excited KD, ab initio calculations reveal an easy‐axis anisotropy for all three cobalt(II) centers, which apparently coincides with the hard axis of magnetization in the ground state KD (see Supporting Information, Figure S11). All results regarding the single‐ion magnetic anisotropy in **Co3P** are in accordance with the previous findings for **Co3C**.^[18]^

Going back to the properties of the trinuclear ensemble, the experimental magnetic susceptibility was also simulated on the basis of the ab initio calculations with the POLY_ANISO program, which employs the Lines model for the magnetic exchange (see Computational Details).^[21]^ The smallest residuals (Supporting Information, Figure S12) between experimental and theoretical magnetic susceptibility data were obtained for slightly different *J* values (*J*_12_/*J*_13_/*J*_23_=−14.2/−17.2/−15.0 cm^−1^). The resulting average value of −15.5 cm^−1^ is close to the experimentally determined value of *J*_ex_=−14.3 cm^−1^. The computed energy gap (8.0 cm^−1^) between the first two KDs of the trinuclear assembly is larger than that previously determined from the experimental data but depends significantly on the asymmetry of the *J* values. The inset in Figure 3 illustrates the orientation of the magnetic anisotropy axes of the ground state KD of **Co3P** (*S*_eff_=1/2 ; *g_x_*=4.15; *g_y_*=2.92; *g_z_*=1.68; see Supporting Information, Table S12 for a full list). The angle of intersection (*g_z_*|Co_3_ plane)=29.0° again significantly depends on the deviation of the *J* values from the trigonal symmetry. The corresponding illustration for the first excited molecular KD can be found in the Supporting Information, Figure S13.

**Figure 3:**
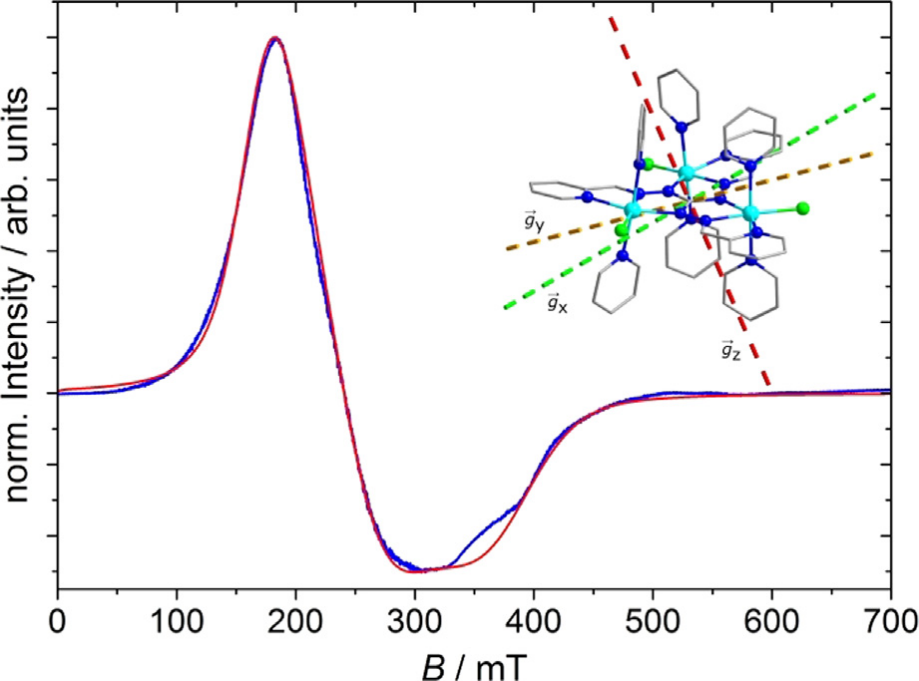
CW X‐band ESR spectrum (blue) of powder **Co3P** recorded at *T=*4.8 K and its corresponding simulation (red) using a simplified single *S*_eff_=1/2 effective spin formalism. Inset: calculated main anisotropy axes (*S*_eff_=1/2 ; based on a POLY_ANISO simulation) for the ground state KD (*g_x_*: easy axis of magnetization; *g_y_*: intermediate axis; *g_z_*: hard axis of magnetization).

CW X‐band ESR experiments at low temperatures on a powdered crystalline sample of **Co3P** were undertaken to experimentally characterize the properties of the magnetic ground state. The ESR spectrum of **Co3P** at 4.8 K (Figure 3) reveals a broad but still very intense signal ranging from 100 to 500 mT. Starting at zero static magnetic field, *B*_0_=0, the signal rises up to a maximum at a value of 170 mT and then decreases to a minimum at 300 mT, featuring two slight shoulders around this minimum. As the broadness of the spectrum does not allow for a precise parametrization, a simulation of the obtained spectrum was performed by keeping the model for the spin system as simple as possible. We employed the single pseudospin *S*_eff_=1/2 formalism, whose validity was corroborated by the fit of the susceptibility data and by ab initio calculations. In first approximation, only one populated KD was assumed to simulate the spectrum at 4.8 K, as mainly the ground state KD should be populated. The simulated ESR spectrum assuming anisotropic line broadenings is depicted in Figure 3, while the used parameters are listed in the Supporting Information, Table S13.

Despite the lack of precision of the determined parameters due to the enormous broadness of the spectrum, the description of the magnetic ground state via a rhombic set of *g* values (*S*_eff_=1/2 ; *g_x_*=3.75; *g_y_*=2.75; *g_z_*=1.85) agrees with the *g* values obtained from the POLY_ANISO simulation (see above and KD1 in the Supporting Information, Table S12) and hence justifies the applied model. A physical interpretation of the signal broadness is the distribution of the single‐ion *g* values due to differences in the cobalt(II) local coordination environments. The minor deviation between the simulated and experimental spectrum around 350 mT can be explained by the additional population of the first excited KD. According to the POLY_ANISO results, this state features very similar *g_x_* and *g_y_* values with respect to the ground state but a *g_z_* value that is beyond the experimental range (Supporting Information, Table S12). When increasing temperature and hence population of the first excited KD, the normalized intensity of the spectrum should decrease at higher magnetic fields. This trend is illustrated in the Supporting Information, Figure S14, where the shape of the spectrum remains unchanged, as expected if a nearly equal population of the first two KDs can be assumed from 10 K upwards.

Taking advantage of the crystal symmetry and packing of **Co3P**, the spin–electric coupling was investigated by the EFM‐ESR technique that was recently developed and employed to study the magnetoelectric effect in molecular helices.^[14]^ The particular aspects of this technique are the application of an oscillating electric field during a CW ESR absorption experiment and the phase‐sensitive detection of the induced signal. Therefore, the experiment is similar to a standard ESR one, but instead of using a modulated static magnetic field *B*_0_, the position of the resonance line and other features such as intensity and linewidth are modulated by the applied electric field *E*_m_. In order to introduce *E*_m_ in the resonating cavity of the ESR spectrometer, a propagating structure without cutoff given by two thin parallel conducting wires at a relative distance of approximately 1 mm was used (Figure 4). An acceptable *E*_m_ homogeneity over the crystal volume is expected in this two‐wire transmission line, also thanks to the polarization charge induced on the sample. The alternating voltage to feed the electrodes was taken from the ESR spectrometer, as it is normally used to drive the modulation coils.

**Figure 4:**
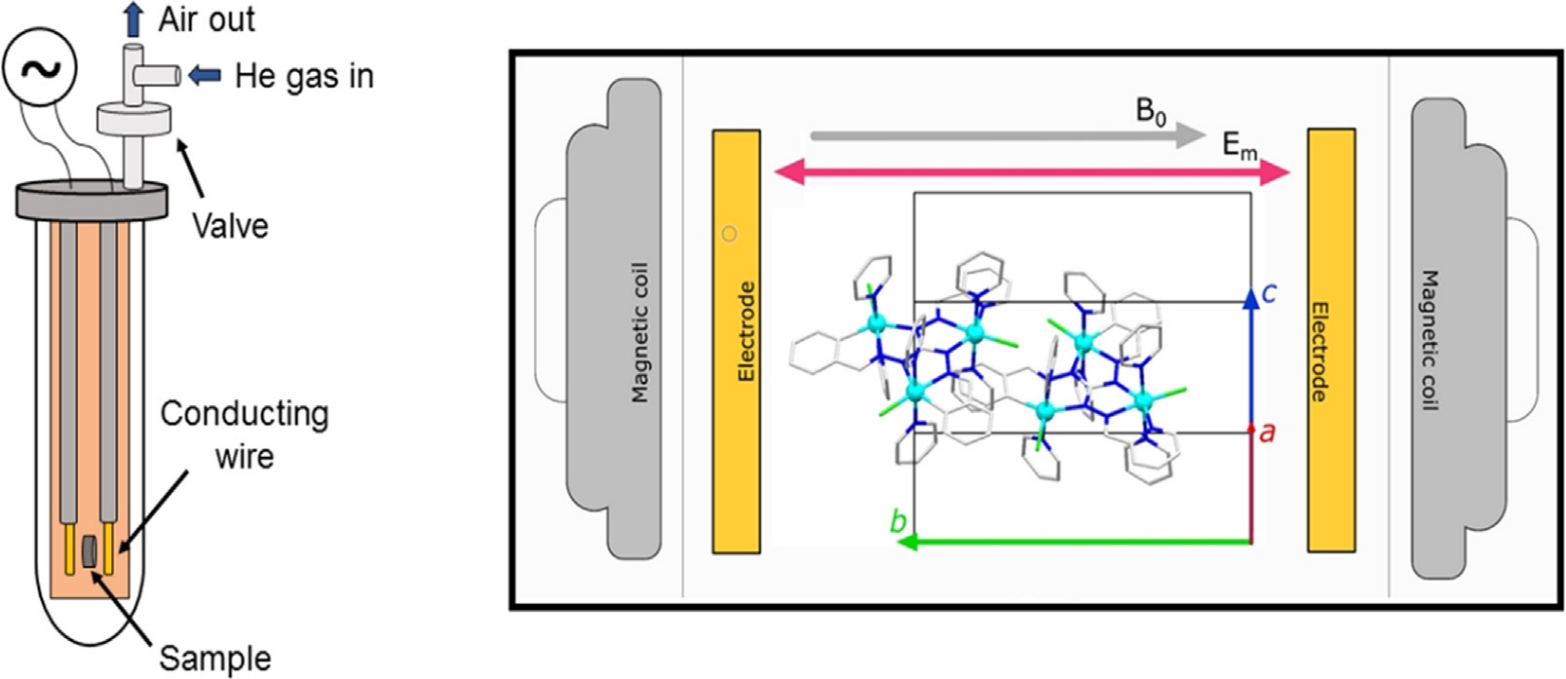
Left: schematic view of the modified version of the sample holder used for the EFM‐ESR and ESR measurements. Right: orientation of the **Co3P** single crystal mounted on the sample holder and placed inside the resonating cavity. The two trinuclear cobalt(II) molecules of the unit cell are shown. Further details are given in the Supporting Information, Figure S15.

In the present case, the faces of a single crystal of **Co3P** were indexed by using a single‐crystal X‐ray diffractometer. The crystal was mounted on the customized sample holder so that the two wire electrodes generate an electric field parallel to the crystallographic $\vec{b}$ axis (Figure 4). The sample holder was then inserted in a quartz ESR tube, filled with He gas, sealed, and placed in the resonating cavity.

The EFM‐ESR spectra were acquired at X‐band (9.4 GHz) with a 30 kHz oscillating *E*_m_ of the order of 15 kV m^−1^. The microwave output power was 100 times higher than that employed to record the standard ESR spectrum. In order to improve the signal‐to‐noise ratio in these measurements, several acquisitions were collected. The reported signal is the sum of all the acquisitions normalized to the number of acquisitions. All spectra were acquired at the constant temperature of 20 K, as a compromise between the lowest temperature achievable and thermal stability, given the heating effect introduced by the modulated electric field. The EFM‐ESR measurements were acquired with the crystal axis $\vec{b}$ parallel to the direction of $\vec{B_{0}}$ and $\vec{E_{m}}$ (Supporting Information, Figure S15). For an individual triangle, this crystal axis nearly coincides with the easy axis of magnetization of the ground state KD (angle of intersection ($\vec{b}$ |*g_x_*)=7.2°, see also Figure 3), as revealed by the ab initio computational studies.

The single‐crystal ESR spectrum at 20 K is shown in Figure 5 (top) and exhibits a broad signal extended over almost the entire measured field range (80–420 mT). This experimental configuration, selected after small rotations of the crystal in the crystallographic *ab* plane, as shown by the spectra given in the Supporting Information, Figure S16, represents an extreme of the ESR signal and shows the smallest linewidth. This guarantees the correct alignment of the $\vec{b}$ along $\vec{B_{0}}$ , because the two molecules give the same signal in this condition. The EFM‐ESR spectra in Figure 5 (bottom) were acquired with $\vec{E_{m}}$ along the direction of $\vec{B_{0}}$ and $\vec{b}$ and evidence a pronounced effect of the applied electric field. In this orientation, the EFM‐ESR spectrum changes sign if the polarity is inverted. This further proves that the EFM‐ESR spectrum is associated with a linear electric field effect. It is also worth noting that in this experimental configuration, the spurious modulated magnetic field induced by $\vec{E_{m}}$ is of the order of 10^−7^ mT,^[14]^ perpendicular to $\vec{B_{0}}$ , and phase shifted by 90° with respect to the modulating applied voltage and, therefore, resulting in a null signal in the phase sensitive detection. Consequently, its contribution to the detected signal can be excluded.

**Figure 5:**
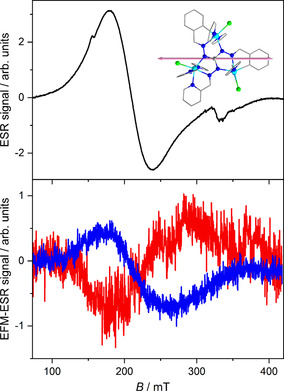
CW X‐band ESR spectrum of a single crystal of **Co3P** with the crystal axis $\vec{b}$ parallel to the direction of $\vec{B_{0}}$ (top) and EFM‐ESR spectra (bottom) acquired with $\vec{E_{m}}$ aligned along the axis $\vec{b}$ at *t*=0 (blue line, resulting from ten acquisitions) and with inverted polarity (red line, resulting from four acquisitions). Inset: molecular structure of the complex cation and the direction of $\vec{b}$ coincident with $\vec{B_{0}}$ and $\vec{E_{m}}$ (pink arrow).

The center of the EFM‐ESR spectra, at *g*≈3.2, roughly coincides with that of the ESR spectrum, while the electric field induced signal is broader (linewidth of ca. 100 mT). The first derivative shape of the spectrum indicates that the dominant effect of the electric field is on the resonance position and is therefore interpreted as an effect on the *g* value. On the contrary, modulation of intensity or linewidth would result in an absorption‐like or 2^nd^‐derivative line shape, respectively. For a linear electric field effect, the spin–electric coupling can be estimated by the ratio between the intensity of the EFM‐ESR and standard ESR spectra.^[11]^ Since the intensity of the ESR spectrum is proportional to the modulation amplitude, we can associate the intensity of the EFM‐ESR signal generated by the modulation of the electric field with an equivalent modulation of the magnetic field.^[11]^ From the estimated value of $B_{m}^{\mathrm{eq}}$ =4.1×10^−4^ mT, the relative shift of the *g* value (Δ*g*_E_) induced by *E*_m_ can be calculated according to Equation 2 directly derived by the resonance condition, where *B*_res_ is the resonant field value in the ESR spectrum.

(2) $$\Delta g_{E}=g\frac{B_{m}^{\mathrm{eq}}}{B_{\mathrm{res}}}$$

Equation (2) gives Δ*g*_E_=1.6×10^−6^. By scaling this effect for the value of the electric field used in the experiment, we obtain Δ*g*_E_/*E=*0.11×10^−9^ m V^−1^. If converted into a frequency shift, this corresponds to approximately 0.3 Hz m V^−1^. The effect is slightly larger than the one reported for the Cu_3_ antiferromagnetic triangle investigated by pulsed ESR spectroscopy in frozen solution.^[13]^ The latter system is characterized by an antiferromagnetic exchange interaction about twenty times stronger than for **Co3P** and by a genuinely frustrated ground state. A spin–electric coupling about one order of magnitude larger than the present one has been recently reported for a Fe_3_ oxo‐centered cluster, again in frozen solution.^[22]^ For the latter, a key role of an unusually large Dzyaloshinskii–Moriya interaction within the triangle was evidenced. The effect found in **Co3P** is comparable with the spin–electric effect observed in Mn‐radical molecular helices.^[14]^ However, considering the broadness of the EFM‐ESR signals, which reflects the broad ESR signals, such an effect cannot be directly observed in a single sweep, which was instead possible in the case of the Mn‐radical helix.

The clear observation of the effect suggests that a significant spin–electric coupling is active in this molecule. Given that the degeneracy of the frustrated state is already removed, as derived from the simulations of the magnetic data, it is not obvious to attribute the observed spin–electric phenomenon to the original effect foreseen by Loss and co‐workers for frustrated triangles.^[6]^ On the other side, we have shown that the low energy spectrum of levels is very sensitive to the imbalance of the *J* values. It is worth to underline that the identification of the dominant mechanism for which a spin–electric effect is visible cannot be a priori attributed to exchange coupling, but requires a more in‐depth investigation. In this respect, the modulation of the magnetic anisotropy and effective *g* values, originating from the strong orbital contribution typical of octahedral cobalt(II) ions, can also be expected to be relevant. Interestingly, mononuclear cases for which strong single‐ion anisotropy promotes spin–electric coupling were reported very recently for a molecular magnet based on a single holmium ion^[16]^ as well as on cerium ions in an inorganic solid matrix.^[17]^ To the best of our knowledge, this is the first quantification of such an effect in a single crystal of a transition‐metal‐based molecular magnet.

In conclusion, the new modification **Co3P** was structurally characterized and its magnetic susceptibility successfully parametrized applying a pseudo‐*C*_3_ symmetry. Theoretical studies revealed slight differences in the magnetic exchange between the individual Co⋅⋅⋅Co pairs present in **Co3P**. Furthermore, the differences in the local magnetic anisotropy for the individual cobalt(II) ions were studied, and an easy‐plane type of magnetic anisotropy with a strong rhombic distortion was found for all three magnetic centers. Such a distortion is influenced by the relative orientation of the pyridine co‐ligands. The local magnetic anisotropy, in combination with the small differences in the individual coupling constants, leads to a lifting of the degeneracy of the first two KDs. This is also reflected in low‐temperature ESR studies probing the molecular magnetic ground state of **Co3P**. The spin–electric coupling revealed by the EFM‐ESR spectra cannot be decisively attributed to a specific mechanism as either the modulation of exchange coupling or magnetic anisotropy may contribute. Our work shows that manipulation of spin by the electric field is feasible also for non‐frustrated triangles. However, deeper comprehension of the mechanisms allowing a sizeable spin–electric coupling is required for a rational design of electrically controllable molecular qubits.

## Conflict of interest

The authors declare no conflict of interest.

## Supporting information

As a service to our authors and readers, this journal provides supporting information supplied by the authors. Such materials are peer reviewed and may be re‐organized for online delivery, but are not copy‐edited or typeset. Technical support issues arising from supporting information (other than missing files) should be addressed to the authors.

Supplementary
