# Supplementary material for: Spin–Electric Coupling in a Cobalt(II)‐Based Spin Triangle Revealed by Electric‐Field‐Modulated Electron Spin Resonance Spectroscopy
Source: Angew Chem Int Ed Engl. 2021 Mar 9;60(16):8832–8. doi: 10.1002/anie.202017116 (PMC8048656; doi:10.1002/anie.202017116)
Supplement: Supplementary file 1 — Supplementary [file ANIE-60-8832-s001.pdf]

Angewandte Chemie
Eine Zeitschrift der Gesellschaft Deutscher Chemiker

Supporting Information

Spin–Electric Coupling in a Cobalt(II)-Based Spin Triangle Revealed by Electric-Field-Modulated Electron Spin Resonance Spectroscopy

Benjamin Kintzel, Maria Fittipaldi, Michael Böhme, Alberto Cini, Lorenzo Tesi, Axel Buchholz, Roberta Sessoli, and Winfried Plass**

anie_202017116_sm_miscellaneous_information.pdf

Supporting Information
©Wiley-VCH 2020
69451 Weinheim, Germany

DOI: 10.1002/anie.2016XXXXX

Table of Contents

	**Experimental Procedures**	S8
Table S1	Crystallographic data and structure refinement parameters	S11
Figure S1	Synthesis scheme of the trinuclear cobalt complex $[Co_3(pytag)(py)_6Cl_3]ClO_4\cdot3py$.	S12
	**Crystal Structure Description**	S12
Figure S2	Illustration of the π···π stacking interactions.	S14
	Selected bond lengths and angles of the coordination environment of the three cobalt(II) centers	S14
**Table S2**		
Table S3	Continuous shape measures	S15
Figure S3	Overlay of the different cationic complex structures $[Co_3(pytag)(py)_6Cl_3]ClO_4$.	S16
Figure S4	Schematic definition of the dihedral *angle* ϑ formed by the aromatic π-planes of the two pyridine co-ligands connected to a cobalt center (depiction: ϑ for Co1 in Co3P).	S17

Table S4	Dihedral angle ϑ (as defined in Figure S4) **formed** by **the** aromatic π-planes of the two pyridine co-ligands for all Co(II) centers in Co3P and Co3C (the latter are taken from ref. [1]; see Figure S3 for an overlay of both cationic complex structures)	S17
	**Magnetic Susceptibility**	S18
Figure S5	Experimental magnetic susceptibility of **Co3P** in the low temperature regime.	S19
Table S5	Obtained parameters by a fit of the temperature dependent magnetic susceptibilty data	S19
Figure S6	Influence of the Euler angle of rotation β.	S20
	**Computational Studies**	S21
Figure S7	Dinuclear cobalt(II) computational model structures used for the BS-DFT calculations.	S21
Table S6	BS-DFT results for **Co3P**	S21

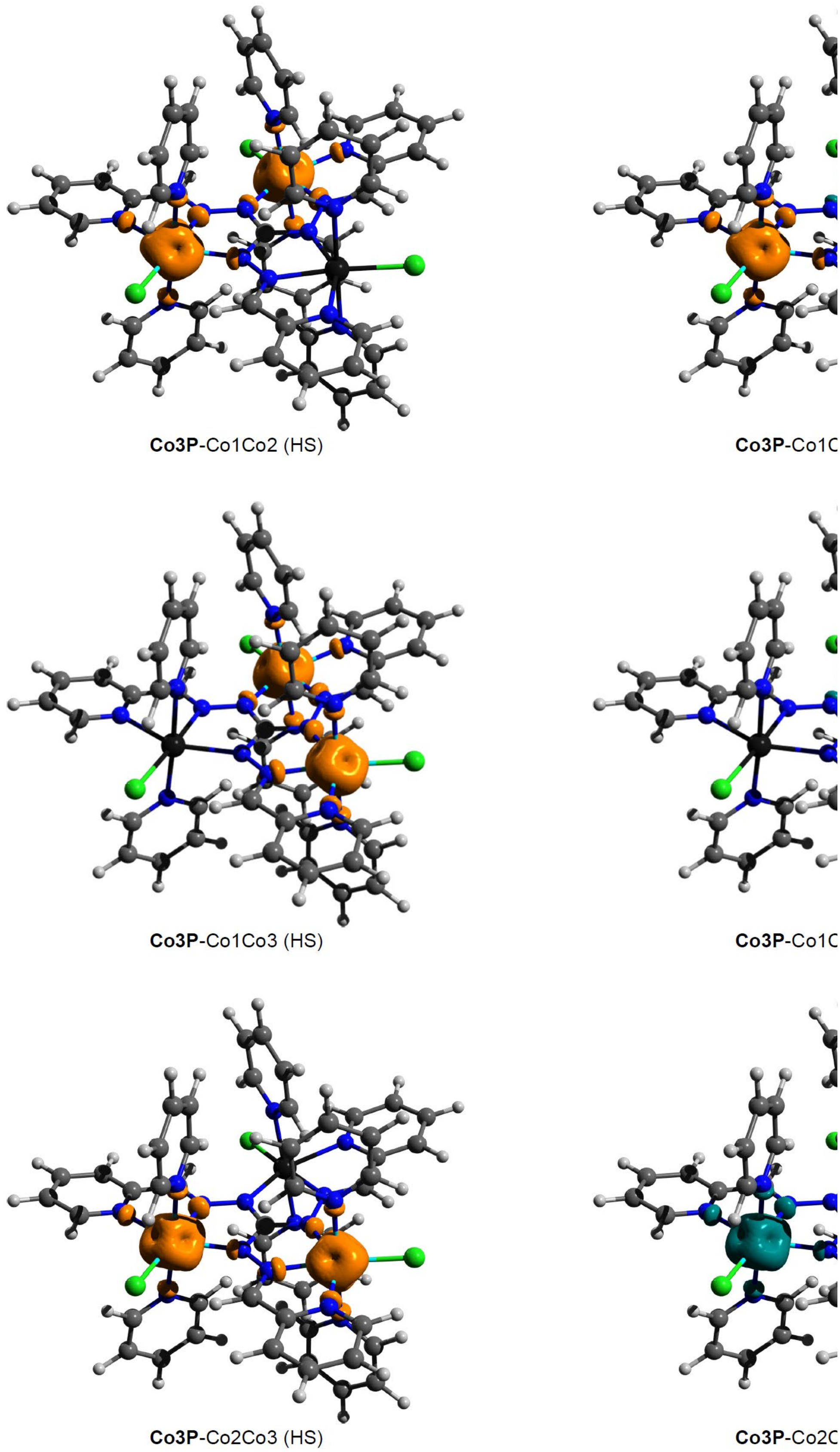

Figure S8 Spin-density isosurfaces for the high-spin and broken-symmetry states of **Co3P**. S22
Table S7 Basis sets used for the *ab initio* calculations of **Co3P** S23

Figure S9	Mononuclear cobalt(II) computational model structures used for the *ab initio* calculations of **Co3P**.	S23
Table S8	Relative CASSCF energies for **Co3P**	S24
Table S9	Relative CASSCF/CASPT2 energies for **Co3P**	S25
Table S10	Relative CASSCF/CASPT2/RASSI-SO energies for **Co3P**	S25
Figure S10	Relative ligand-field splitting for the three cobalt(II) centers in **Co3P**.	S26
Table S11	Cartesian components of the *g* factor in **Co3P**-Co1, **Co3P**-Co2, and **Co3P**-Co3	S26
Figure S11	Ab initio calculated (S_{eff} = ½) easy-axis anisotropy for the first excited KD.	S20
Figure S12	POLY_ANISO simulation of the magnetic susceptibility of **Co3P** based on the *ab initio* calculations.	S28
Table S12	Cartesian components of the *g* factor for the first four KDs (S_{eff} = ½).	S28
Figure S13	Calculated main anisotropy axes (S_{eff} = ½) for the first excited molecular Kramers doublet in **Co3P**.	S29
	**ESR Studies**	S29
Table S13	Parameters used for simulations of the powder CW X-band ESR spectra	S29

Figure S14 Temperature dependence of normalized CW X-band ESR spectrum. S30
Schematic view of the EFM-ESR set-up S31

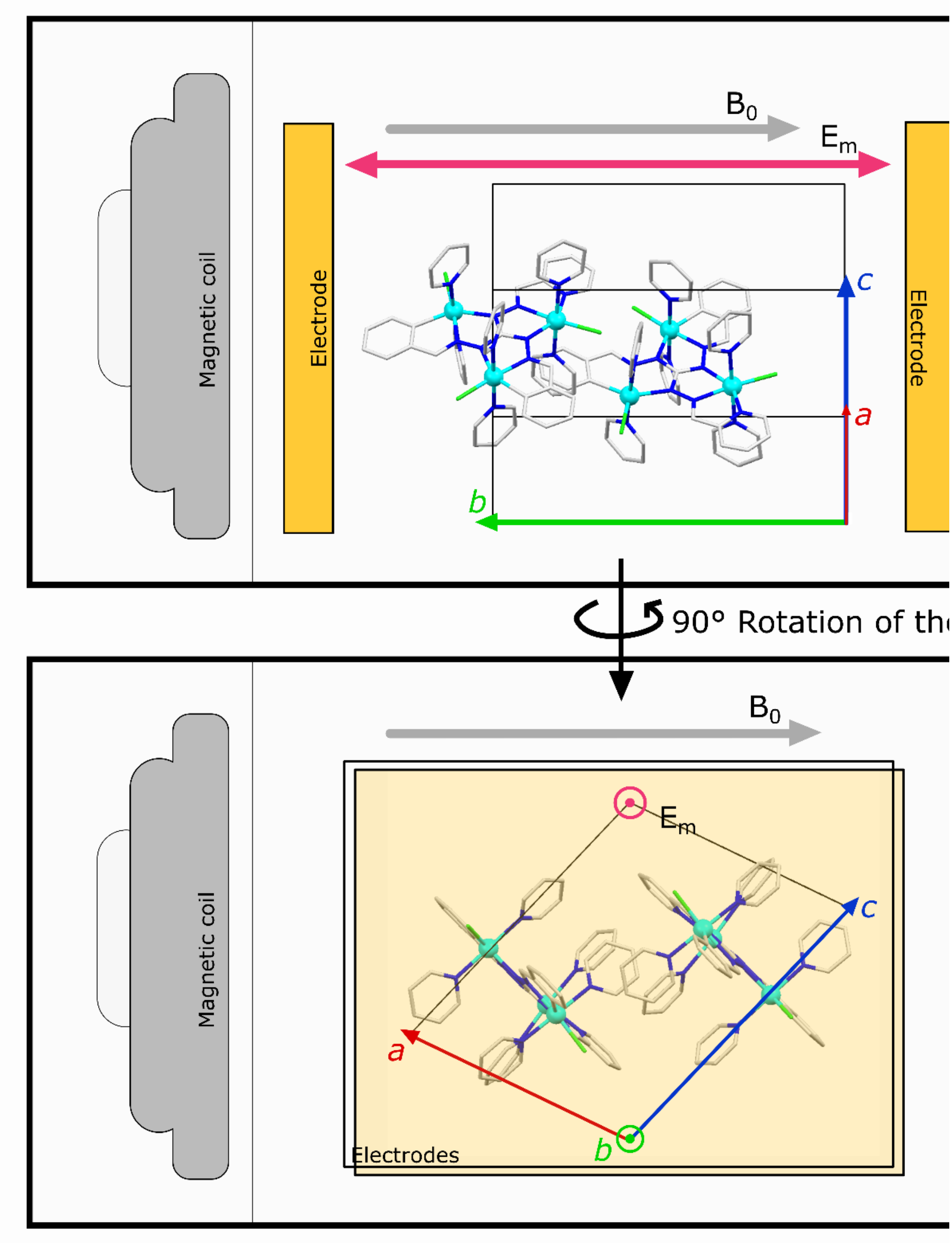

Figure S15 Single Crystal X-Band CW-ESR spectra of a single crystal of Co3P acquired at different orientations of the $\vec{b}$ S32

axis in the *ab* plane: $\vec{b}$ parallel to the direction of B_0 (black line), $\vec{b}$ at an angle of 10° with respect to B_0 (blue line) and $\vec{b}$ at an angle of 20° with respect to B_0 (red line).

Supplementary References S3

Experimental Procedures

Materials. Cobalt(II) salts were used as purchased in synthesis grade from commercial sources, all solvents were distilled prior to use, pyridine for complex synthesis was distilled over CaH_2 under N_2 atmosphere. The monohydrochloride of the chelate ligand (H_2pytag·HCl) was synthesized as described in the literature.[1] ***Caution!*** *Perchlorate salts of metal complexes containing organic ligands are potentially explosive. Only a small amount of material should be prepared, and it should be handled with great care.*

Synthesis of [Co₃(pytag)(py)₆Cl₃]ClO₄·3py (Co3P). (see also

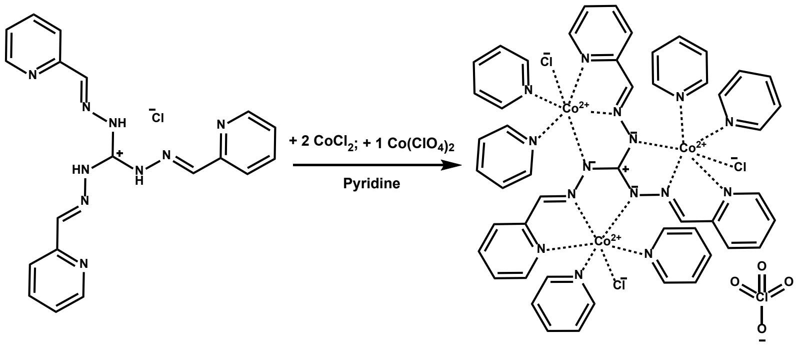

Figure S1) All operations were carried out in standard Schlenk technique under N_2 inert atmosphere. $CoCl_2·6H_2O$ (128 mg, 0.536 mmol) and $Co(ClO_4)_2·6H_2O$ (98 mg, 0.268 mmol) were jointly dissolved in pyridine (15 mL) and H_2pytag·HCl (122 mg, 0.268 mmol) was added as a solid. The mixture was stirred for 5 minutes and subsequently filtered through a G3 frit. The filtrate was left unmoved for at least four weeks at room temperature, which allows for the growth of pure phase large block crystals of **Co3P**. The mother liquor was decanted and the crystals were washed with ice-cold pyridine followed by decantation for two times. The crystals were then dried by means of a N_2 stream. Yield: 170 mg (0.116 mmol, 43 %)

Elemental analysis: calculated for $C_{64}H_{60}Cl_4Co_3N_{18}O_4$ ([Co_3(pytag)(py)$_6Cl_3$]ClO_4·3py): C 52.51 %, H 4.13 %, N 17.22 %. Found: C 52.05 %, H 4.11 %, N 17.00 %. **IR**: (ATR) $\tilde{\nu}$ [cm^{-1}] = 3067 (w), 2998 (w), 1598(m), 1439 (m), 1393 (s), 1339 (s), 1138 (s), 1090 (s broad, ClO_4), 700 (s), 621 (s).

For all experiments exclusively the described intact crystalline material was used, which can be handled under ambient conditions for several minutes without noteworthy chemical decomposition or wear of the crystallographic cell, which was checked by repeated cell determinations by X-ray diffractometry. When not under investigation, crystals were stored in Nujol oil under N_2 atmosphere or at −60° C under N_2 atmosphere or in a liquid N_2 cooling container, in doing so the chemical and crystallographic intactness could be retained.

Elemental analysis. The elemental analysis was measured on a EURO EA CHNSO Analyser by HEKAtech and a VARIO EL III Analyser by Elementar Analysensysteme.

Infrared spectroscopy. IR-spectra were recorded on an FT-IR spectrometer of the Type Vertex 70 by Bruker. The solid samples were placed on a Golden-Gate-ATR unit by Specac for measurement.

Crystal Structure Determination. Single-crystal X-ray diffraction studies were carried out with a Bruker D8 Venture diffractometer equipped with IµS 3.0 microfocus X-ray source (Cu-K_α, λ = 1.54178 Å), PHOTON III detector and fixed-Chi/Kappa Goniometer. The data were collected at a fixed temperature of 100(2) K and used to solve the structure of **Co3P** (see Table S1 for full details). For this, a clear light red prism-like specimen with approximate dimensions 0.1 mm × 0.1 mm × 0.2 mm was used. The Bruker software package SAINT was used for data reduction and cell refinements. Data were corrected for absorption effects using the Multi-Scan method (SADABS). The structure was solved and refined using the Bruker SHELXTL software package and using the full-matrix least-squares refinement on F^2. Deposition Number 2051871 contains the supplementary crystallographic data for this paper. These data are provided free of charge by the joint Cambridge Crystallographic Data Centre and Fachinformationszentrum Karlsruhe Access Structures service www.ccdc.cam.ac.uk/structures.

Magnetic Susceptibility Measurements. A single crystal of **Co3P** was ground and placed in a gelatine capsule. Magnetic measurements were performed on a Quantum Design MPMS-5 SQUID magnetometer. Susceptibility data were obtained in the temperature range from 2 to 300 K at an applied dc field H_{dc} of 1000 Oe. The collected data were corrected for the diamagnetism of the sample holder, the capsule, and the diamagnetic contribution of the ligand. The fitting of the magnetic susceptibilty data with full matrix diagonalization was carried out using the program PHI in version 3.1.5.[2]

Single Crystal and Powder CW X-Band ESR. A single crystal of **Co3P** was placed on a square acetate foil with Apiezon N vacuum grease and indexed with an SCD Oxford Xcalibu3 X-Ray diffractometer using a Cu source (Cu-K$_\alpha$, λ = 1.54060 Å). Once the crystallographic orientations of the crystal were known, the crystal was mounted on an ESR support for single crystals and measured. For powder measurements, a single crystal of **Co3P** was ground and filled in an ESR tube. The measurements were performed on a Bruker E580 in an MS-5 resonator at 4.8 K with an exact microwave frequency of 9.4 GHz. Simulations of the obtained spectra were carried out using EasySpin.[3]

Single Crystal Electric Field-Modulated X-Band ESR. For the experiments, a well-shaped single crystal of **Co3P** was picked and indexed on an X-ray diffractometer (same procedure as described for ESR measurements), so that the crystal could be positioned on the sample holder as described. The electric field modulated (EFM)-ESR measurements were performed with the setup as described in the literature.[4] EFM-ESR spectra were acquired with 100 times higher microwave power with respect to the ESR spectra. In order to improve the signal-to-noise ratio in the EFM-ESR measurements, several acquisitions were realized. The reported signal is the sum of all the acquisitions normalized to root square of the number of acquisition. All spectra were acquired at 20 K. The EFM-ESR measurements were acquired with the crystal axis $\vec{b}$ parallel to the direction of E_m and at 0° with respect to the direction of B_0.

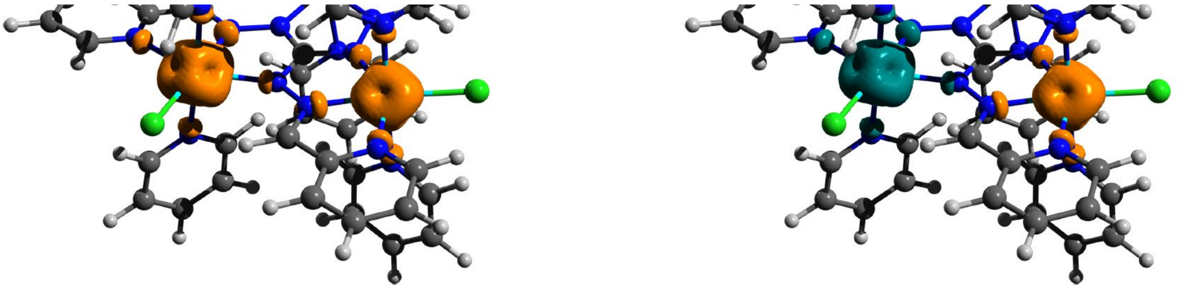

Co3P-Co2Co3 (HS) **Co3P**-Co2Co3 (BS)

ystal t RI- ıc(II) tions ɔuta- ɗef2- ama-

Figure S8).

$$J = \frac{2(E_{BS}-E_{HS})}{\langle S^2_{HS}\rangle - \langle S^2_{BS}\rangle} \quad (S1)$$

Single-ion anisotropies for the three crystallographically independent cobalt(II) ions in **Co3P** were calculated with the OpenMolcas package of programs in version 18.09 at CASSCF/CASPT2/SO-RASSI level of theory and the basis sets listed in Table S7.[12] For these calculations three mononuclear cobalt(II) model structures in which the two remaining paramagnetic ions have been replaced by diamagnetic zinc(II) ions. The mononuclear *ab initio* model structures are denoted as **Co3P**-Co1, **Co3P**-Co2, and **Co3P**-Co3, respectively, and visualized in Figure S9. State-average CASSCF calculations contained the 7 electrons of the 3d shell in 10 orbitals (3d and 4d shell) to adequately take the so-called 'double d-shell effect' into account[13] and were performed for 10 quartet (^{4}F, ^{4}P) and 40 doublet states (^{2}G, ^{2}P, ^{2}H, ^{2}D, ^{2}D, ^{2}F). Dynamic correlation was treated by the CASPT2 approach on the base of the CASSCF wave functions for all quartet and the 12 lowest doublet states. Subsequently, RASSI-SO calculations on the basis of the CASPT2 wave functions were employed to consider spin–orbit coupling which allows a mixing of states including those of different multiplicities. On the basis of the RASSI-SO wave functions, single-ion anisotropies were obtained by the SINGLE_ANISO module of OpenMolcas and molecular properties of the trinuclear complex cation were calculated using the POLY_ANISO program[14] by simulating molecular magnetic properties based on the ab initio calculations combined with three individual magnetic exchange constants (J_{12}, J_{13}, and J_{23}) based on the Lines model (see Computational studies for values).[15]

Table S1. Crystallographic data and structure refinement parameters for $[Co_3(pytag)(py)_6Cl_3]ClO_4{\cdot}3py$ (**Co$_3$P**)

empirical formula	$C_{64}H_{60}Cl_4Co_3N_{18}O_4$
CCDC number	2051871
formula weight	1463.89
crystal system	monoclinic
space group	$P2_1$ (no. 4)
a / pm	1223.86(16)
b / pm	1760.8(2)
c / pm	1604.7(2)
α / °	90
β / °	107.740(7)
γ / °	90
V / nm^3	3.2936(8)
Z	2
T / K	100(2)
λ / Å	1.54178
ρ / g·cm^{-3}	1.476
F(000)	1502
abs. coefficient / mm^{-1}	7.851
Θ range of data collection / °	$2.89 \leq \Theta \leq 72.40$
measured reflections	29857
unique reflections (R_{int})	9241 (0.0622)
reflections [$I > 2\sigma(I)$]	7625
goodness of fit on F^2	1.054
Flack parameter	0.009(7)
final R indices [all data]	R_1 = 0.0714, ωR_2 = 0.1452
final R indices [$I > 2\sigma(I)$]	R_1 = 0.0558, ωR_2 = 0.1271

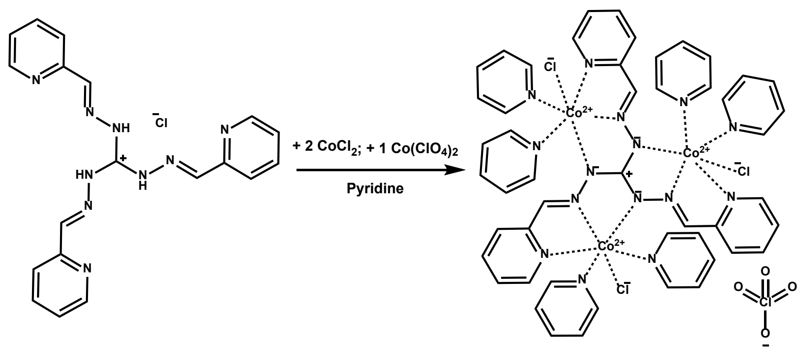

Figure S1. Synthesis scheme of the trinuclear cobalt complex $[Co_3(pytag)(py)_6Cl_3]ClO_4 \cdot 3py$ (**Co$_3$P**).

Crystal Structure Description

Intermolecular π···π stacking interactions can be observed in **Co3P** and are formed by the 2-pyridyl moiety of the chelate ligand, as is illustrated in Figure S2. This interconnects the trinuclear complex cations along the crystallographic $\vec{a}$ axis in a layered staircase-like manner. The respective distances between the π-planes show an average value of approximately 360 pm, which is a reasonable magnitude for π···π stacking interactions. As a consequence, the closest intermolecular Co···Co distance is found to be 815.1 pm. The π-plane of the tritopic ligand $pytag^{2-}$ features a minor bowl-shaped distortion, the terminal 2-pyridyl moieties protrude from the central triaminoguanidine plane. Therefore, the three pyridine co-ligands 'in the bowl' (the ones with donor atoms N10–N12) are packed closer than the ones on the opposite site of tritopic ligand (co-ligands with donor atoms N13–N15). Interestingly, the aromatic planes of the pyridine co-ligands 'in the bowl' in all three cases align along the direction of the respective Co–Cl bond, while the remaining other ones do not appear to have a preferential alignment.

Selected bond lengths and angles of the coordination environment for the three crystallographically independent cobalt(II) centers in **Co3P** are listed in

Table S2. The coordinative bond lengths range from 210.0 (Co1–N2) to 236.7 pm (Co1–Cl1), where the distances to the chlorido donors, which are weak and large in ionic radius, are always longest as is expected. A rather narrow distribution of bond lengths is observed for the remaining Co–N distances, tending to be above 220 pm for the axial pyridine donors and below 220 pm for the equatorial chelate ligand donors. The neighboring donor angles reach from 73° (N2–Co1–N4) to 112.6° (N1–Co3–Cl3), which indicates a significant distortion due to the high deviation from the ideal octahedron case of 90°. The three cobalt(II) centers within one cationic complex molecule are bridged by two neighboring N–N diazine moieties. The intramolecular Co···Co distances are 517.9 pm (Co1···Co2), 513.9 pm (Co1···Co3), and 513.0 pm (Co2···Co3).

Overall, the bond lengths and angles within the coordination spheres of the cobalt(II) centers are comparable to the ones in the structure of **Co3C**.[1] To further characterize and compare the distortion of the coordination geometry of the distinct cobalt(II) centers, continuous shape measures for **Co3P** have been undertaken.[16–18] The latter reveal deviation parameters from an ideal octahedron of $S(O_h)$ = 2.514, 2.230, and 2.256 for Co1, Co2, and Co3, respectively ($S(O_h)$ = 0 refers to an ideal octahedron). Those are by far the smallest deviation parameters in comparison to any other ideal coordination geometry given in Table S3. Hence, all three coordination polyhedra in **Co3P** can be regarded as moderately distorted octahedra, although atom Co1 shows a slightly higher distortion

than the other two metal centers. Looking at the deviation from the average of all three values (**Co3P**: 2.333; **Co3C**: 2.402), the distortion of the coordination spheres is a bit more reduced and homogeneous in the case of **Co3P** ($S(O_h)$ range in **Co3P**/**Co3C**: 2.230-2.514/2.196-2.695). The corresponding continuous shape measures for **Co3C** are also given in Table S3.

An overlay representation of the complex cation $[Co_3(pytag)(py)_6Cl_3]^+$ with the one found in **Co3C** is presented in Figure S3. It reveals only minor differences between the cationic complex molecules in **Co3P** and **Co3C**, for which the orientation of the axial pyridine co-ligands can be determined as the largest structural difference.

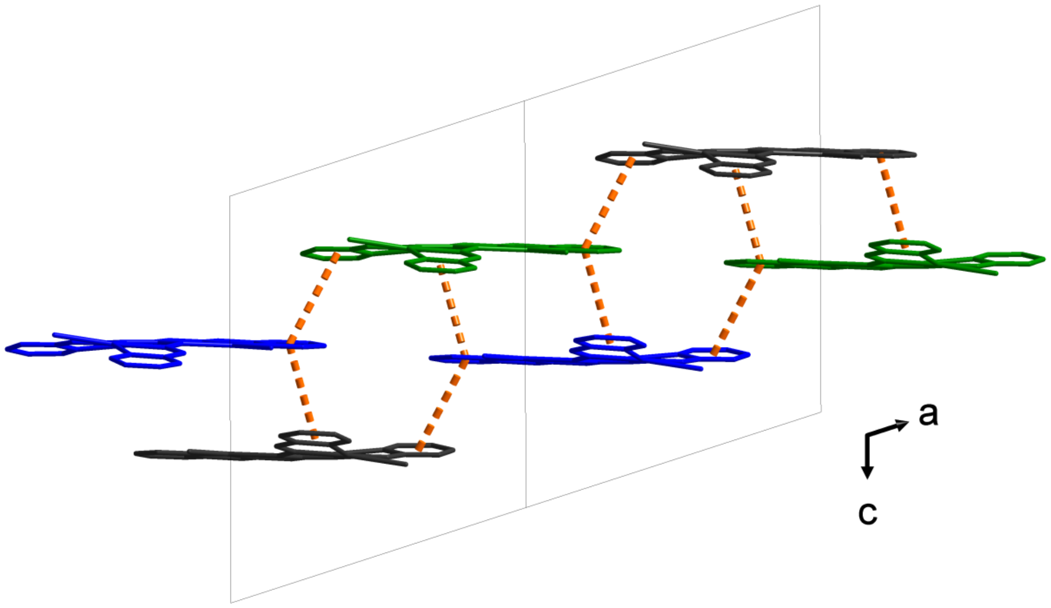

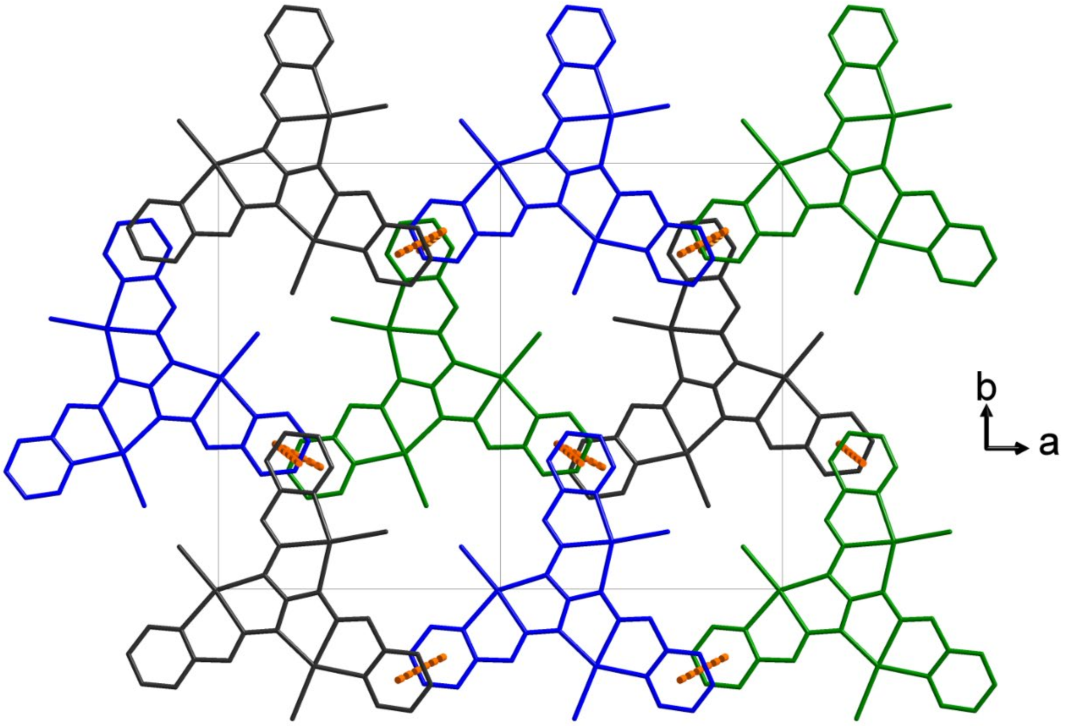

Figure S2. Illustration of the π···π stacking interactions in **Co3P**, which are emphasized by orange dashed bonds between the centroids of the involved aromatic rings. A staircase-like structure is formed along the crystallographic $\vec{a}$ axis. Top: View along the crystallographic $-\vec{b}$ axis, the cationic complex molecules including two unit cells along the *a* axis. Bottom: View along the crystallographic $-\vec{c}$ axis of the same ensemble of molecules. Hydrogen atoms, perchlorate anions, co-crystallized solvent molecules, and the axial pyridine co-ligands have been omitted for clarity. The color code of the molecules represents their relative position along the crystallographic $\vec{c}$ axis.

Table S2. Selected bond lengths (pm) and angles (°) of the coordination environment of the three cobalt(II) centers within the trinuclear complex cation of **Co3P**

Co1−N2	210.0(7)	Co2−N5	213.7(8)	Co3−N8	211.3(8)
Co1−N4	213.6(7)	Co2−N7	212.9(7)	Co3−N1	211.3(7)
Co1−N3	216.2(8)	Co2−N6	214.1(7)	Co3−N9	216.8(7)
Co1−N10	221.5(7)	Co2−N11	224.4(7)	Co3−N12	224.1(7)
Co1−N13	217.2(7)	Co2−N14	223.6(7)	Co3−N15	220.8(7)
Co1−Cl1	236.7(2)	Co2−Cl2	235.2(3)	Co3−Cl3	234.8(3)

N2−Co1−N4	73.0(3)	N5−Co2−N7	72.8(3)	N8−Co3−N1	73.6(3)
N2−Co1−N3	75.3(3)	N5−Co2−N6	75.3(3)	N8−Co3−N9	75.2(3)
N2−Co1−N10	93.2(3)	N5−Co2−N11	91.1(3)	N8−Co3−N12	91.0(3)
N2−Co1−N13	86.1(3)	N5−Co2−N14	87.8(3)	N8−Co3−N15	89.6(3)
N2−Co1−Cl1	172.6(2)	N5−Co2−Cl2	175.0(2)	N8−Co3−Cl3	173.7(2)
N3−Co1−N4	147.4(3)	N6−Co2−N7	148.0(3)	N9−Co3−N1	148.8(3)
N3−Co1−N10	86.0(3)	N6−Co2−N11	89.3(3)	N9−Co3−N12	91.9(3)
N3−Co1−N13	94.8(3)	N6−Co2−N14	87.1(3)	N9−Co3−N15	88.4(3)
N3−Co1−Cl1	100.1(2)	N6−Co2−Cl2	100.6(2)	N9−Co3−Cl3	98.6(2)
N4−Co1−N10	88.5(3)	N7−Co2−N11	90.1(3)	N1−Co3−N12	88.3(3)
N4−Co1−N13	90.3(3)	N7−Co2−N14	92.9(3)	N1−Co3−N15	91.7(3)
N4−Co1−Cl1	112.19(18)	N7−Co2−Cl2	111.4(2)	N1−Co3−Cl3	112.6(2)
N10−Co1−N13	178.8(3)	N11−Co2−N14	176.3(3)	N12−Co3−N15	179.4(3)
Cl1−Co1−N10	92.27(19)	Cl2−Co2−N11	91.7(2)	Cl3−Co3−N12	90.5(2)
Cl1−Co1−N13	88.58(18)	Cl2−Co2−N14	89.1(2)	Cl3−Co3−N15	88.9(2)

Table S3. Continuous shape measures for the metal ions in the two complexes **Co3P** and **Co3C** (a value S = 0 describes an ideal polyhedron; OC−6 = octahedron; TPR−6 = trigonal prism; PPY−6 = pentagonal pyramid; JPPY−6 = Johnson pentagonal pyramid; HP−6 = Hexagon) [16–18]

		OC–6 $S(O_h)$	TPR–6 $S(D_{3h})$	PPY–6 $S(C_{5v})$	JPPY–6 $S(C'_{5v})$	HP–6 $S(D_{6h})$
**Co3P**	Co1	2.514	11.224	22.036	25.596	33.124
	Co2	2.230	13.448	22.924	26.076	32.939
	Co3	2.256	12.707	22.974	26.297	32.650
**Co3C**	Co1	2.196	12.907	23.405	26.809	33.172
	Co2	2.695	10.509	22.087	25.581	32.928
	Co3	2.315	11.327	22.734	26.105	32.808

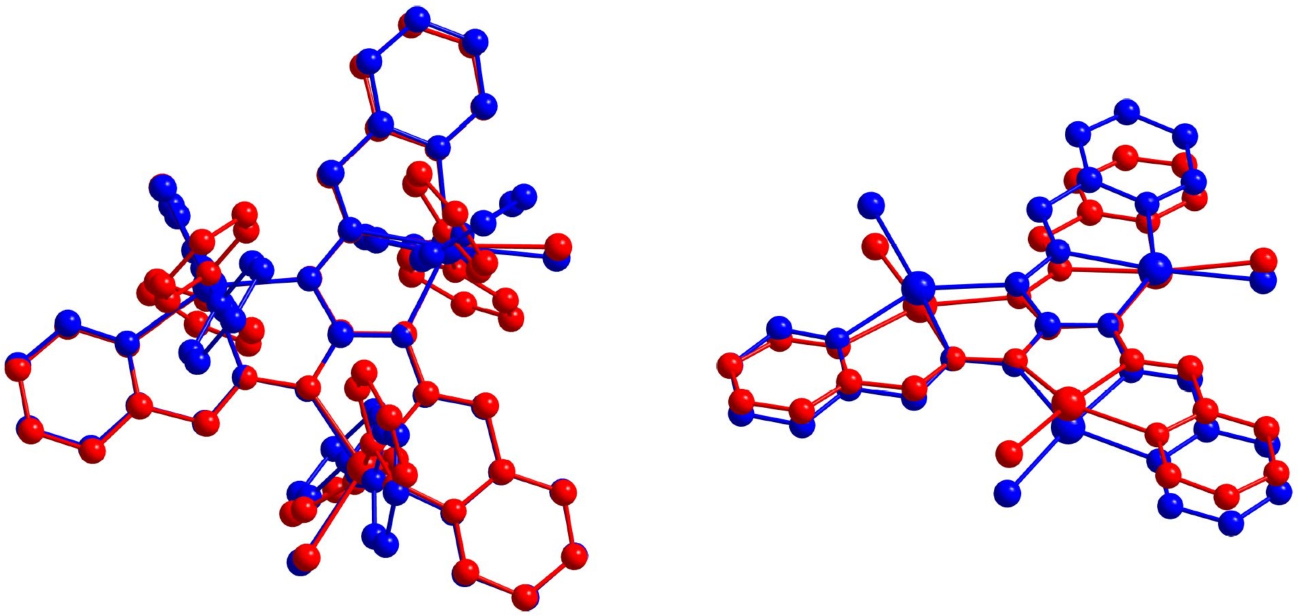

Figure S3. Overlay of the cationic complex structure $[Co_3(pytag)(py)_6Cl_3]^+$ as found in **Co3P** (blue) and **Co3C** (red). The figure on the left-hand side shows a top-view perspective and the right-hand side displays the triaminoguanidine-based ligand backbone with the axial pyridine co-ligands removed. Hydrogen atoms have been omitted for clarity.

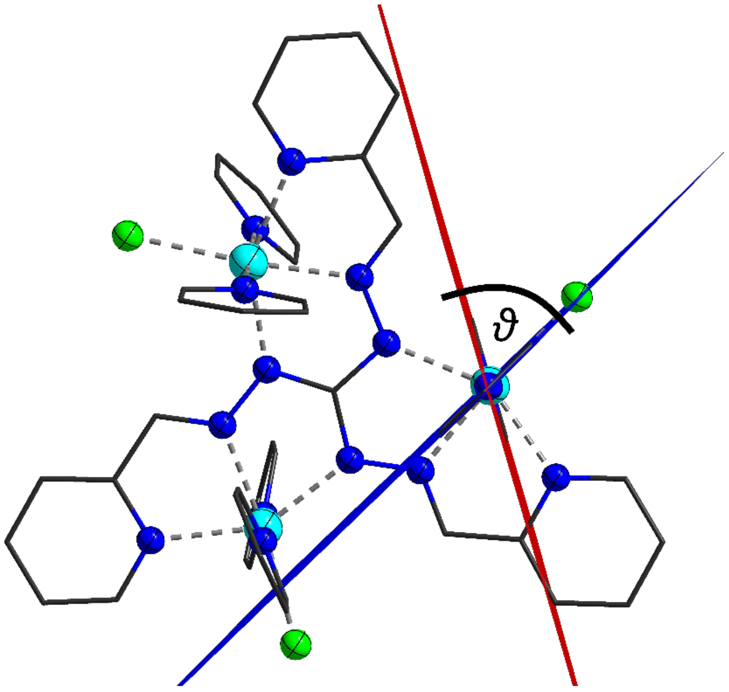

Figure S4. Schematic definition of the dihedral angle ϑ formed by the aromatic π-planes of the two pyridine co-ligands connected to a cobalt center (depiction: ϑ for Co1 in **Co3P**).

Table S4. Dihedral angle ϑ (as defined in

Figure **S4**) formed by the aromatic π-planes of the two pyridine co-ligands for all Co(II) centers in **Co3P** and **Co3C** (the latter are taken from ref. [1]; see Figure S3 for an overlay of both cationic complex structures)

ϑ	**Co3P**	**Co3C**
Co1	61.7°	27.8°
Co2	36.5°	68.6°
Co3	44.0°	27.8°

Magnetic Susceptibility

Description of the Hamiltonian used for fitting and simulation of the magnetic susceptibility

In the following part the single terms of the used Hamiltonian given in the main manuscript are shown and described in detail. In case of the magnetic exchange as defined in Equation (S2), a single isotropic coupling constant $-J_{ex}$ has been used in the model to avoid a potential overparametrization, which assumes an equilateral triangle spin topology for **Co3P**.

$$\mathbf{H_{ex}} = -J_{ex}(\mathrm{S_1S_2 + S_1S_3 + S_2S_3}) \quad \text{(S2)}$$

The spin-orbit contribution as represented by Equation (S3) includes the spin-orbit coupling constant for an octahedrally coordinated cobalt(II) ion in a weak ligand-field (λ = −171.5 cm^{-1}).[19] The orbital-reduction parameter λ is a fit parameter (2/3 < λ < 1) and due to the structural similarity between all three cobalt(II) centers, again, has been condensed to a single parameter for all three ions.

$$\mathbf{H_{SO}} = -\frac{3}{2}\kappa\lambda\sum_{i=1}^{3}\mathrm{S}_i\mathrm{L}_i \quad \text{(S3)}$$

The ligand-field splitting (LFS) of the three individual cobalt(II) centers in **Co3P** has been described by two extended Stevens operators ($\mathrm{O}_2^0 = 3\mathrm{L}_z - \mathrm{L}^2; \mathrm{O}_2^2 = \frac{1}{2}[\mathrm{L}_+^2 + \mathrm{L}_-^2]$) and the corresponding LFS parameters B_2^0 and B_2^2, respectively (Equation (S4)).[20] Based on the first LFS parameter, different types of magnetic anisotropy in the ground state KD can be described (B_2^0 < 0: easy axis; B_2^0 > 0: easy plane; B_2^0 = 0: isotropic). The second LF parameter B_2^2 introduces a rhombic distortion of the magnetic anisotropy ($B_2^2 \neq 0$). In addition, an Euler rotation (*zxz*' convention) as represented by the rotation matrix $\overline{\mathrm{R}}^{(\alpha_i,\beta)}$ is included to take the local magnetic anisotropy into account. The Euler angles of rotation α_i are pre-determined due to an assumed C_3 pseudo-symmetry in the fitting model (α_1 = 0°; α_2 = 120°; α_3 = 240°). The Euler angle of rotation β describes the angle of intersection between the local magnetic *z* axis and the main rotational axis of the C_3 pseudo-symmetry. A third Euler angle of rotation γ has not been applied, i.e. no rotation about the *z*' axis (γ = 0°).

$$\mathbf{H_{LF}} = -\frac{3}{2}\kappa\theta_2\sum_i^3\overline{\mathrm{R}}^{(\alpha_i,\beta)}\left[B_2^0\left(3\mathrm{L}_{z,i} - \mathrm{L}_i^2\right) + \frac{B_2^2}{2}\left(\mathrm{L}_{+,i}^2 + \mathrm{L}_{-,i}^2\right)\right] \quad \text{(S4)}$$

The Zeeman effect for **Co3P** was taken into account by Equation (S5), in which μ_B is the Bohr magneton. The spin contribution includes the Landé factor of the free electron (g_0 = 2.0023) and the orbital contribution is influenced by the orbital reduction factor κ.

$$\mathbf{H_{Ze}} = \mu_B\sum_{i=1}^{3}\left(g_0\mathrm{S}_i - \frac{3}{2}\kappa\mathrm{L}_i\right)\vec{B} \quad \text{(S5)}$$

Single-ion properties in terms of the relative ligand-field splitting of the $^4T_{1g}[^4F]$ multiplet for the three cobalt(II) centers can be described by the combination of spin-orbit contribution and ligand-field splitting as given in Equation (S6).

$$\mathbf{H_{SI}} = \mathbf{H_{SO}} + \mathbf{H_{LF}} \quad \text{(S6)}$$

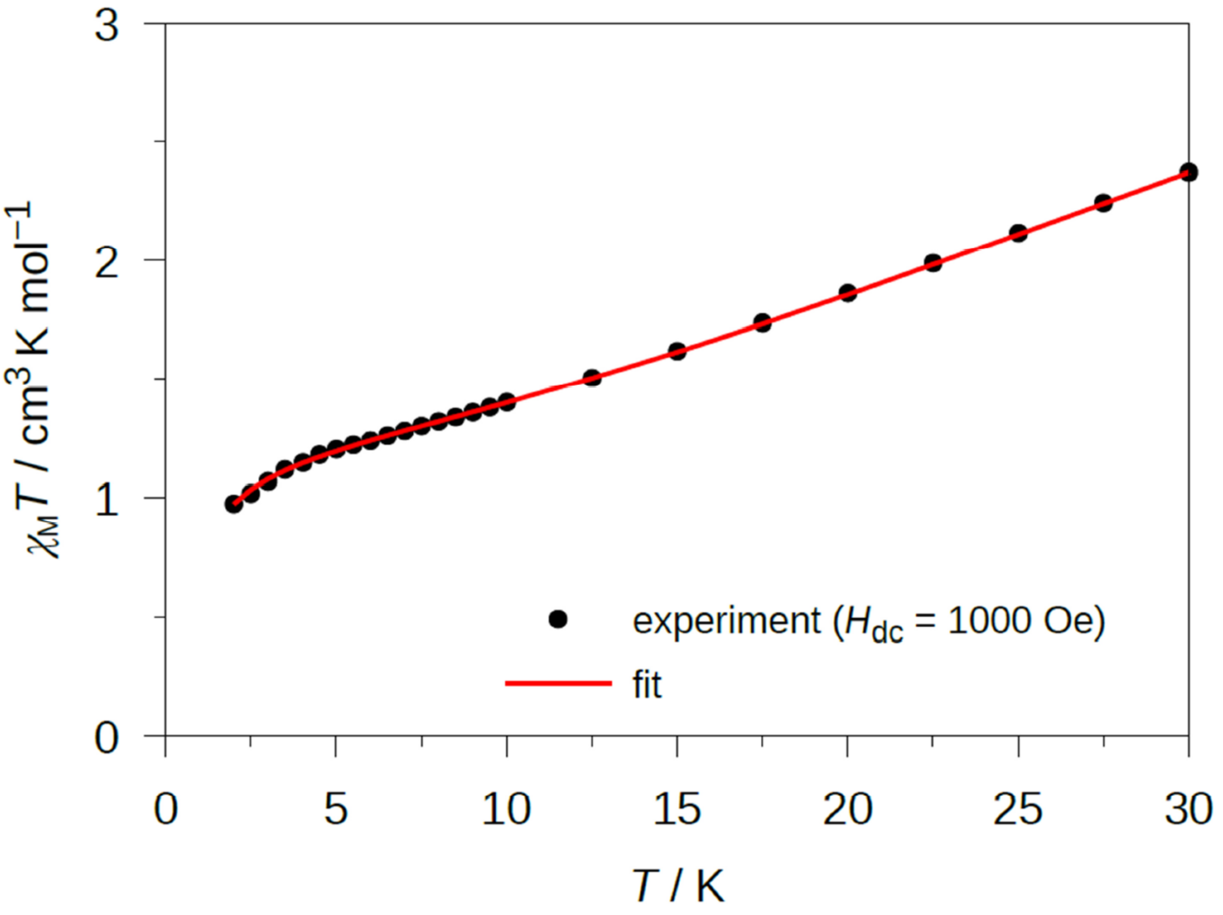

Figure S5. Experimental magnetic susceptibility (●) of **Co3P** in the low temperature regime between 2 and 30 K at an applied dc magnetic field of 1000 Oe. The solid red line shows the best-fit according to the Hamiltonian given in Equation (1) from the main manuscript, fitting parameter values are given in Table S55.

Table S5. Obtained parameters by a fit of the temperature dependent magnetic susceptibility data of **Co3P** at H_{dc} = 1000 Oe according to the Hamiltonian given in Equation (1) from the main manuscript with the particular contributions from the effects described in Equations (S2)–(S5)

parameter	value	value fixed	remark
J_{ex}	−14.3 cm^{-1}	no	
κ	0.99997	no	
B_2^0	151.8 cm^{-1}	no	
B_2^2	−23.1 cm^{-1}	no	
β	20.6°	no	
α_i	0° (i = 1)	yes	angles fixed due to C_3 symmetry constraints
	120° (i = 2)	yes	
	240° (i = 3)	yes	

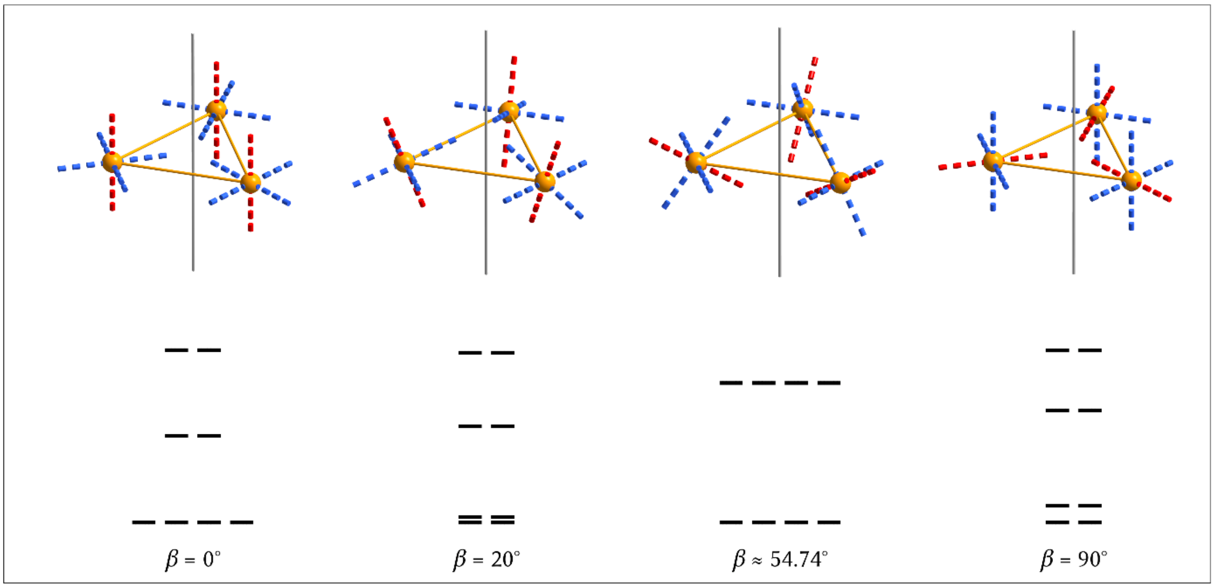

Figure S6. Simulation of the influence of the Euler angle of rotation *β* on the local magnetic axes as well as on the relative energy of the first eight low-lying spin-coupled states (assuming a local easy-plane type of magnetic anisotropy with an identical antiferromagnetic exchange between spin centers; color code: orange spheres − cobalt(II) ions; grey solid line − main rotational axis; red dashed line − local hard axis of magnetization; blue dashed lines − local easy axes of magnetization forming the easy plane).

Computational Studies

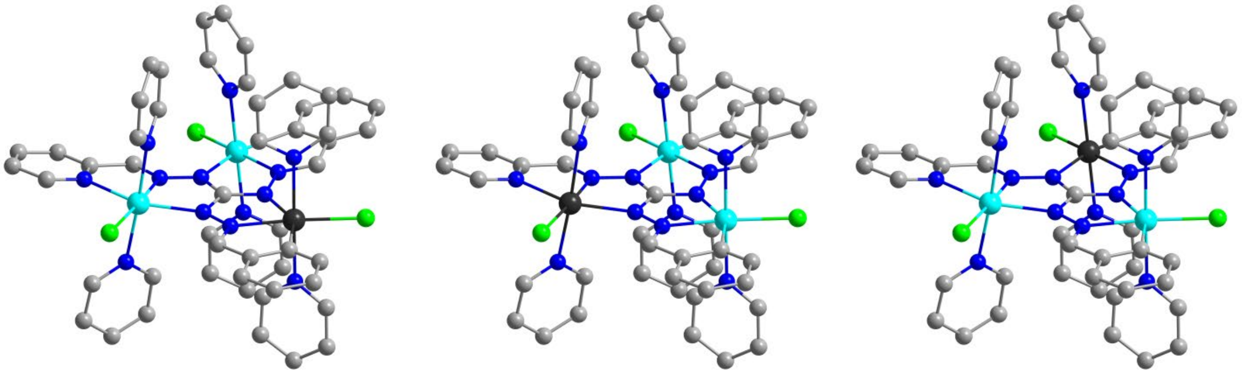

Figure S7. Dinuclear cobalt(II) model structures with the formula $[Co_2Zn(saltag)(Cl)_3(py)_6]^+$ used for the BS-DFT calculations of **Co3P** (color code: Co – cyan; Zn – dark gray). Hydrogen atoms have been omitted for clarity.

Table S6. BS-DFT results for **Co3P** in terms of energies, spin expectation values $\langle S^2 \rangle$, and obtained magnetic coupling constants *J* according to Equation (S1)

Computational model	State (2*S*+1)	E_{DFT}/ Hartree	$\langle S^2 \rangle$	J / cm^{-1}
**Co3P**-Co1Co2	HS (7)<br>BS (1)	−8640.327417<br>−8640.327803	12.023<br>3.019	−18.8
**Co3P**-Co1Co3	HS (7)<br>BS (1)	−8640.325992<br>−8640.326325	12.023<br>3.019	−16.2
**Co3P**-Co2Co3	HS (7)<br>BS (1)	−8640.323255<br>−8640.323613	12.023<br>3.019	−17.5
		$J_{av.} = (J_{12} + J_{13} + J_{13})/3 =$		−17.5

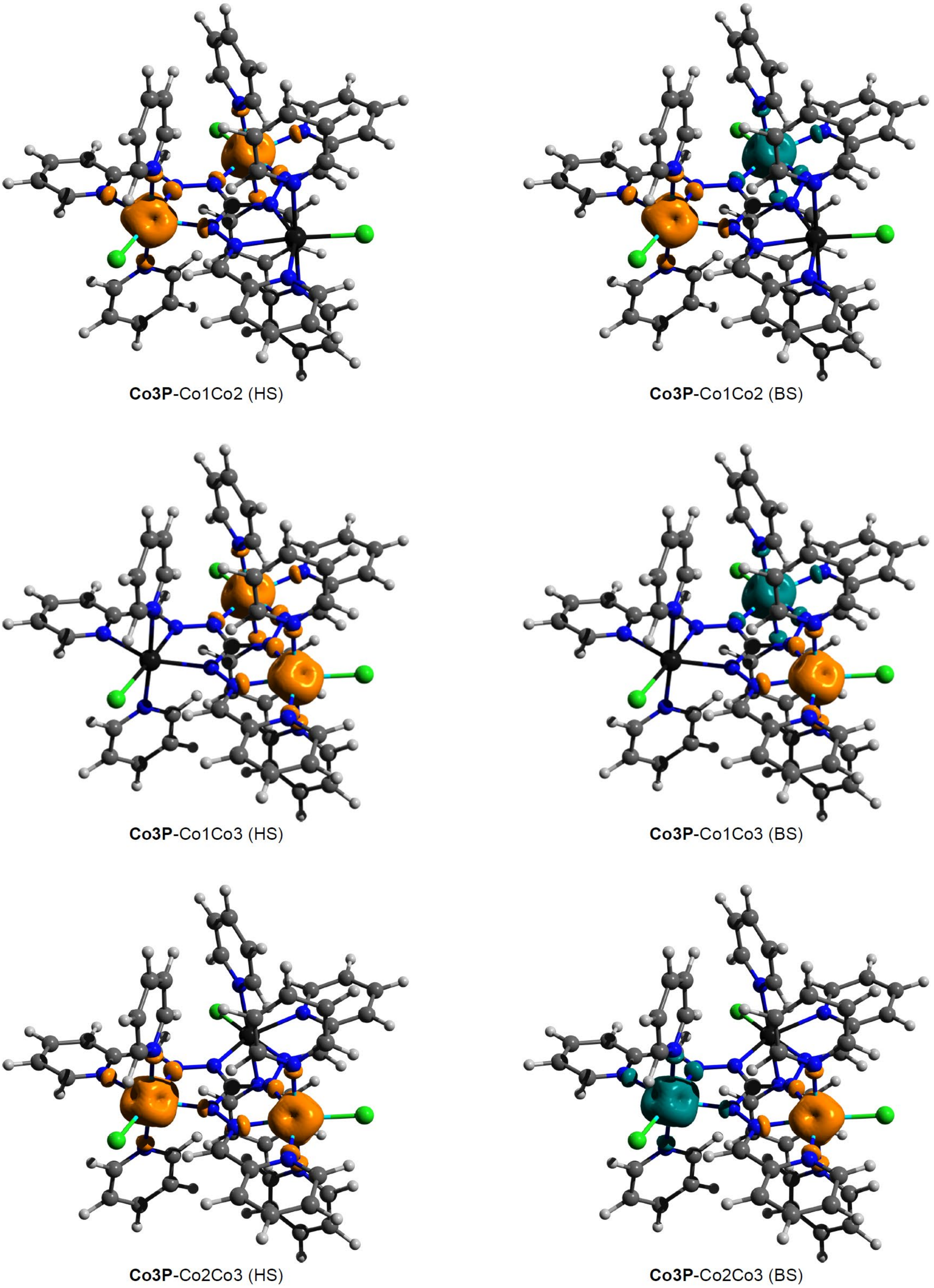

Figure S8. Spin-density isosurfaces (0.01 au; orange = net α density; turquoise = net β density) for the high-spin (left column) and broken-symmetry (right column) states of **Co3P**-Co1Co2, **Co3P**-Co1Co3, and **Co3P**-Co2Co3.

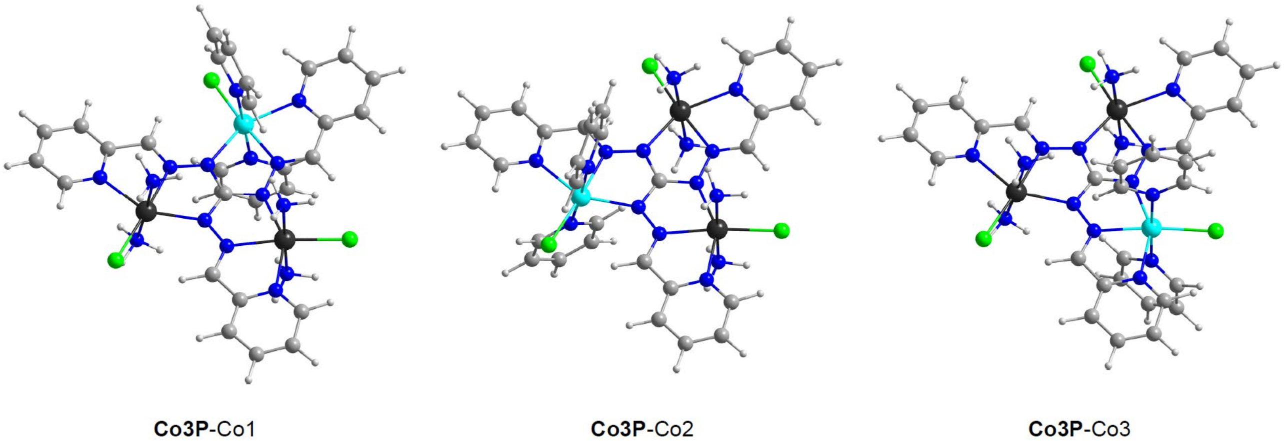

Figure S9. Mononuclear cobalt(II) model structures with the formula $[CoZn_2(saltag)(Cl)_3(py)_2(NH_3)_4]^+$ used for the *ab initio* calculations of **Co3P** (color code: Co – cyan; Zn – dark gray).

Table S7. Basis sets used for the *ab initio* calculations of **Co3P**

Atom type	Basis set	Basis set alias
Co	Co.ANO-RCC...6s5p4d2f1g	ANO-RCC-VTZP
Zn	Zn.ANO-RCC...5s4p2d	ANO-RCC-VDZ
Cl (donor to Co)	Cl.ANO-RCC...5s4p2d1f	ANO-RCC-VTZP
Cl (remaining)	Cl.ANO-RCC...4s3p	ANO-RCC-VDZ
N (donor to Co)	N.ANO-RCC...4s3p2d1f	ANO-RCC-VTZP
N (remaining)	N.ANO-RCC...3s2p	ANO-RCC-VDZ
C	C.ANO-RCC...3s2p	ANO-RCC-VDZ
H	H.ANO-RCC...2s	ANO-RCC-VDZ

Table S8. Relative CASSCF energies (in cm^{-1}) for all quartet and the 12 lowest doublet states of **Co3P**-Co1, **Co3P**-Co2, and **Co3P**-Co3

2*S*+1	Term	Subterm	**Co3P**-Co1	**Co3P**-Co2	**Co3P**-Co3
4	^4F	$^4T_{1g}$	0	0	0
			965	248	335
			1098	516	683
		$^4T_{2g}$	7242	6947	7065
			7985	7269	7478
			8355	7330	7688
		$^4A_{2g}$	16141	15241	15598
	^4P	$^4T_{1g}$	22808	22274	22253
			23808	23038	23137
			23820	23115	23710
2	^2G + ^2P		13621	13326	13276
			14232	14140	14089
			19466	19243	19220
			19981	19538	19607
			20031	19673	19814
			20318	19890	19950
			20467	20011	20221
			20924	20274	20350
			25413	24841	25034
			25445	25047	25129
			25686	25224	25199
			25920	25332	25548

Table S9. Relative CASSCF/CASPT2 energies (in cm^{-1}) for all quartet and the 12 lowest doublet states of **Co3P**-Co1, **Co3P**-Co2, and **Co3P**-Co3

2*S*+1	Term	Subterm	**Co3P**-Co1	**Co3P**-Co2	**Co3P**-Co3
4	^4F	$^4T_{1g}$	0	0	0
			1098	422	462
			1319	645	744
		$^4T_{2g}$	8207	8123	8271
			8749	8427	8765
			9366	8514	8903
		$^4A_{2g}$	18330	17517	17904
	^4P	$^4T_{1g}$	19955	19752	19506
			21074	20728	20492
			21409	20948	21723
2	^2G + ^2P		10766	10467	10593
			11541	11477	11583
			16641	16485	16561
			16982	16890	17172
			17288	17129	17239
			17552	17308	17387
			17735	17417	17738
			18354	17770	17931
			22000	23110	23369
			22820	21100	21282
			21847	21414	21532
			22036	21589	21819

Table S10. Relative CASSCF/CASPT2/RASSI-SO energies (in cm^{-1}) for the six lowest Kramers doublets (KDs) of **Co3P**-Co1, **Co3P**-Co2, and **Co3P**-Co3

Term	Subterm	KD	**Co3P**-Co1	**Co3P**-Co2	**Co3P**-Co3
^4F	$^4T_{1g}$	1	0	0	0
		2	97	200	205
		3	1056	599	570
		4	1273	938	888
		5	1617	1125	1158
		6	1743	1230	1228

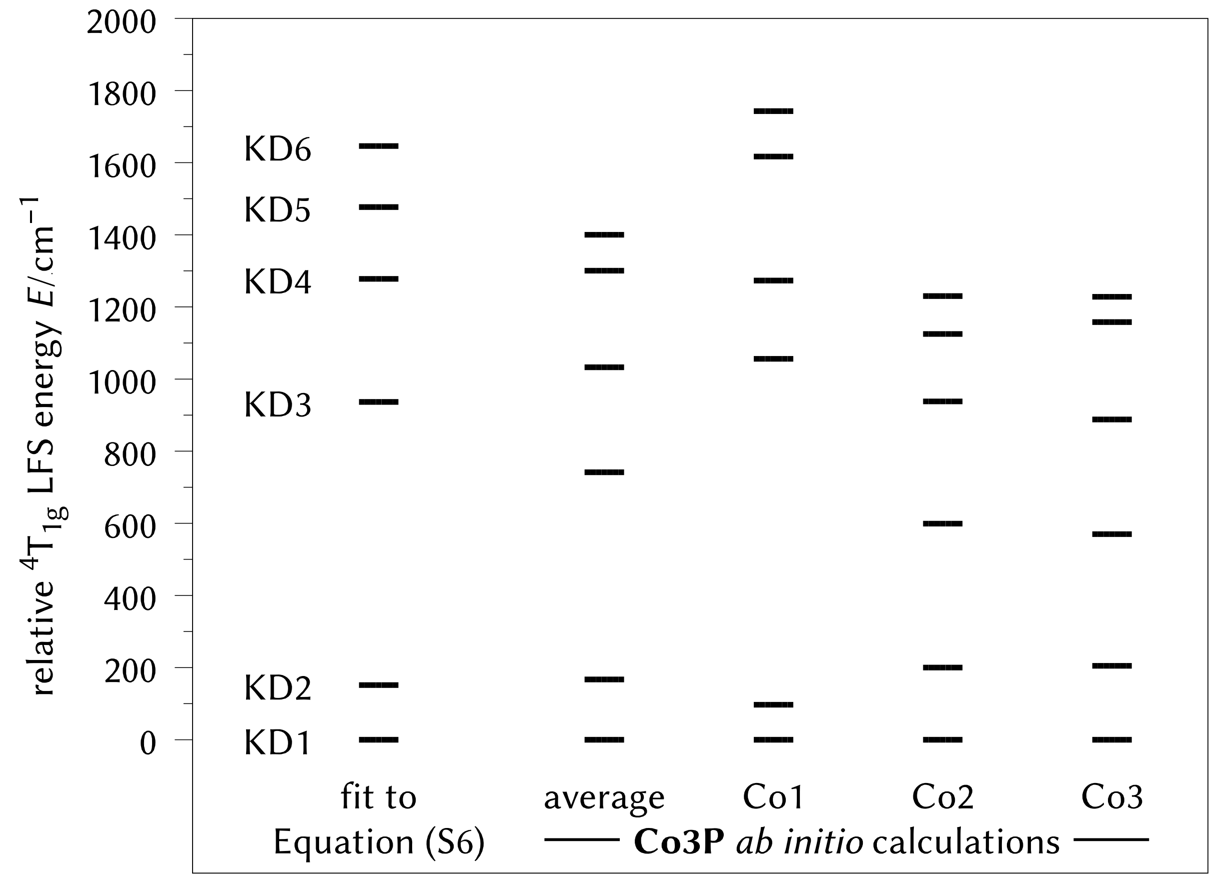

Figure S10. Relative ligand-field splitting of the $^4T_{1g}$ [^4F] multiplet for the three cobalt(II) centers in **Co3P** as obtained by Equation (S6) in combination with best-fit parameters of the magnetic data (left-hand side; κ = 1.00, $\boldsymbol{B_2^0}$ = 151.8 cm^{-1}, $\boldsymbol{B_2^2}$ = −23.1 cm^{-1}) and by *ab initio* calculations (right-hand side).

Table S11. Cartesian components of the *g* factor for the first two Kramers doublets in **Co3P**-Co1, **Co3P**-Co2, and **Co3P**-Co3 (S_{eff} = ½; $g_{av} = \sqrt{(g_x^2 + g_y^2 + g_z^2)/3}$; $g_{xy,av} = \sqrt{(g_x^2 + g_y^2)/2}$) combined with values obtained from a simulation of a single cobalt(II) together with the single-ion anisotropy parameters (κ, $\boldsymbol{B_2^0}$, and $\boldsymbol{B_2^2}$) derived from the best-fit of the experimental values (for values see Table S55)

		**Co3P**-Co1	**Co3P**-Co2	**Co3P**-Co3	Simulation
KD1	g_x	5.441	6.203	6.011	5.267
	g_y	4.466	4.081	4.252	4.735
	g_z	2.139	2.424	2.386	2.148
	g_{av}	4.247	4.510	4.469	4.273
	$g_{xy,av}$	4.977	5.250	5.206	5.008
KD2	g_x	0.421	0.785	0.681	0.234
	g_y	0.475	1.023	0.849	0.235
	g_z	6.118	5.380	5.215	5.540
	g_{av}	3.551	3.194	3.076	3.218
	$g_{xy,av}$	0.449	0.912	0.770	0.234

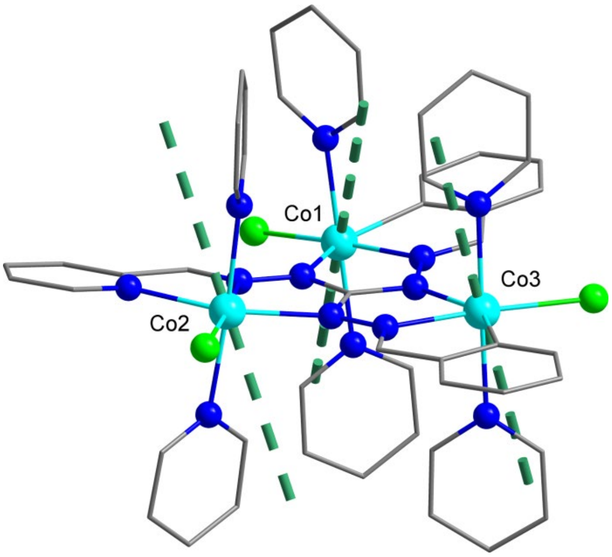

Figure S11. *Ab initio* calculated (S_{eff} = ½) easy-axis anisotropy (green dashed lines) for the first excited Kramers doublet of the three individual cobalt(II) centers in **Co3P** (see also Table S11). Hydrogen atoms have been omitted for clarity.

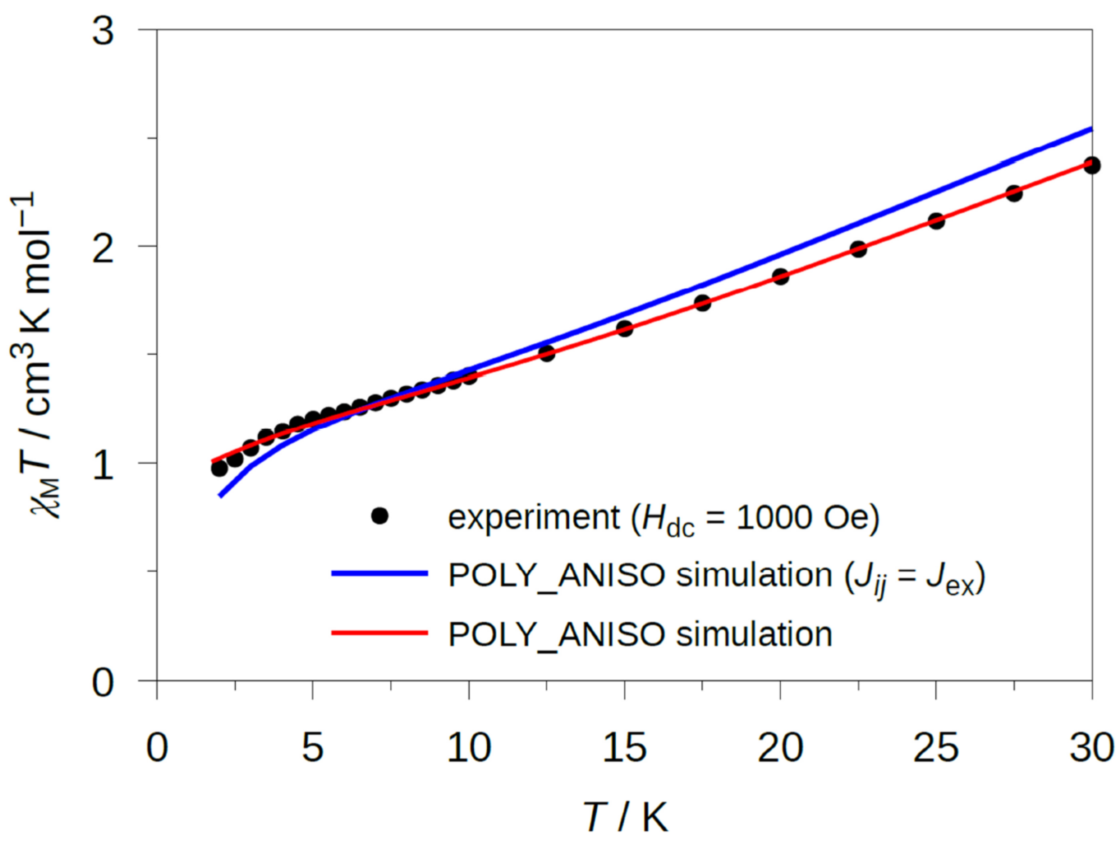

Figure S12. Experimental magnetic susceptibility (●) of **Co3P** in the low-temperature regime between 2 and 30 K at an applied dc magnetic field of 1000 Oe. The solid blue line shows a POLY_ANISO simulation based on the *ab initio* calculations and the experimental coupling constant J_{ex} used for all three individual spin–spin interactions. The solid red line shows a corresponding POLY_ANISO simulation with three individual magnetic coupling constants (J_{12} / J_{13} / J_{23} = −14.2 / −17.2 / −15.0 cm^{-1}) obtained by minimizing the least-square root between experimental and theoretical magnetic susceptibility data.

Table S12. Cartesian components of the *g* factor for the first four KDs (S_{eff} = ½) in the trinuclear cationic complex **Co3P** (values obtained by the POLY_ANISO program employing the *ab initio* calculations combined with three individual coupling constants J_{12} = −14.2 cm^{-1}, J_{13} = −17.2 cm^{-1}, and J_{23} = −15.0 cm^{-1}; $g_{av} = \sqrt{(g_x^2 + g_y^2 + g_z^2)/3}$)

State	Relative energy / cm^{-1}	g_x	g_y	g_z	g_{av}
KD1	0.0	4.148	2.924	1.677	3.086
KD2	8.0	3.933	2.908	0.593	2.845
KD3	40.1	1.002	1.441	6.785	4.046
KD4	68.5	5.746	3.730	1.055	4.002

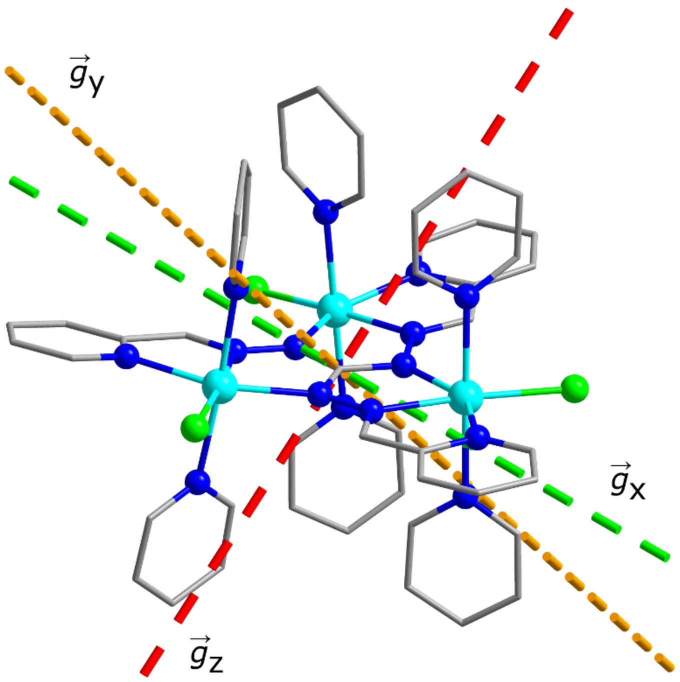

Figure S13. Calculated main anisotropy axes (S_{eff} = ½; based on a POLY_ANISO simulation of the *ab initio* data of the individual single-ions) for the first excited molecular Kramers doublet at 8.0 cm^{-1} of the cationic trinuclear cobalt(II) complex in **Co3P** (g_x: easy axis of magnetization; g_y: intermediate axis; g_z: hard axis of magnetization). Hydrogen atoms are omitted for clarity.

ESR Studies

Table S13. Overview of parameters used for simulations of the powder CW X-band ESR spectra of **Co3P** measured at T = 4.8 K for a pseudospin S_{eff} = ½ formalism to represent the coupled ground state of the whole molecule with one set of g values (isotropic Voigtian (lwpp: Gaussian, Lorentzian [mT]) as well as anisotropic line broadening (Hstrain) were used; for information on the implementation of the parameters see the EasySpin documentation)[3]

S_{eff}	½
lwpp	10, 20
$Hstrain_x$ / MHz	2000
$Hstrain_y$ / MHz	2700
$Hstrain_z$ / MHz	2000
g_x	3.75
g_y	2.75
g_z	1.85

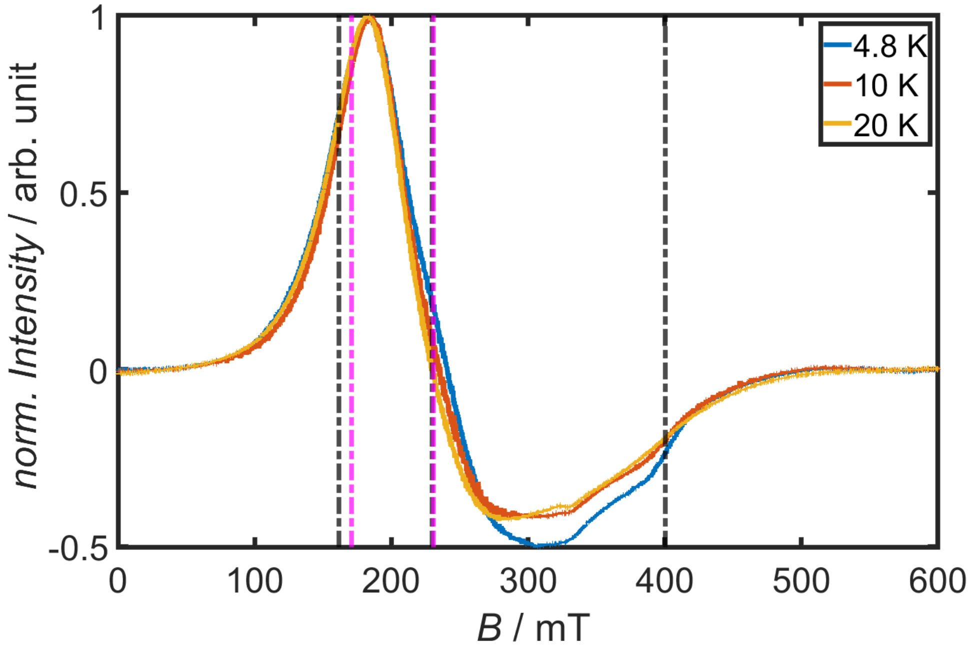

Figure S14. Temperature dependence of normalized CW X-band ESR spectra of **Co3P**. The shape of the spectra does not show further change above 10 K, which is indicative for a nearly equal population of the two lowest lying molecular magnetic states KD1 and KD2. The resonance positions of *ab initio* (POLY_ANISO) calculated Cartesian components of the *g* values (see Table S12) of KD1 (grey) and KD2 (magenta) are marked with dashed lines. The relative decrease of the normalized ESR signal at magnetic fields above 300 mT is in accordance with the computational result of g_z of the second Kramers doublet (KD2) being 0.593 and thus, out of the experimental range. Note that the *ab initio* calculations appear to slightly overestimate the g_x and g_y values as well as underestimate the g_z value of the first Kramers doublet (KD1).

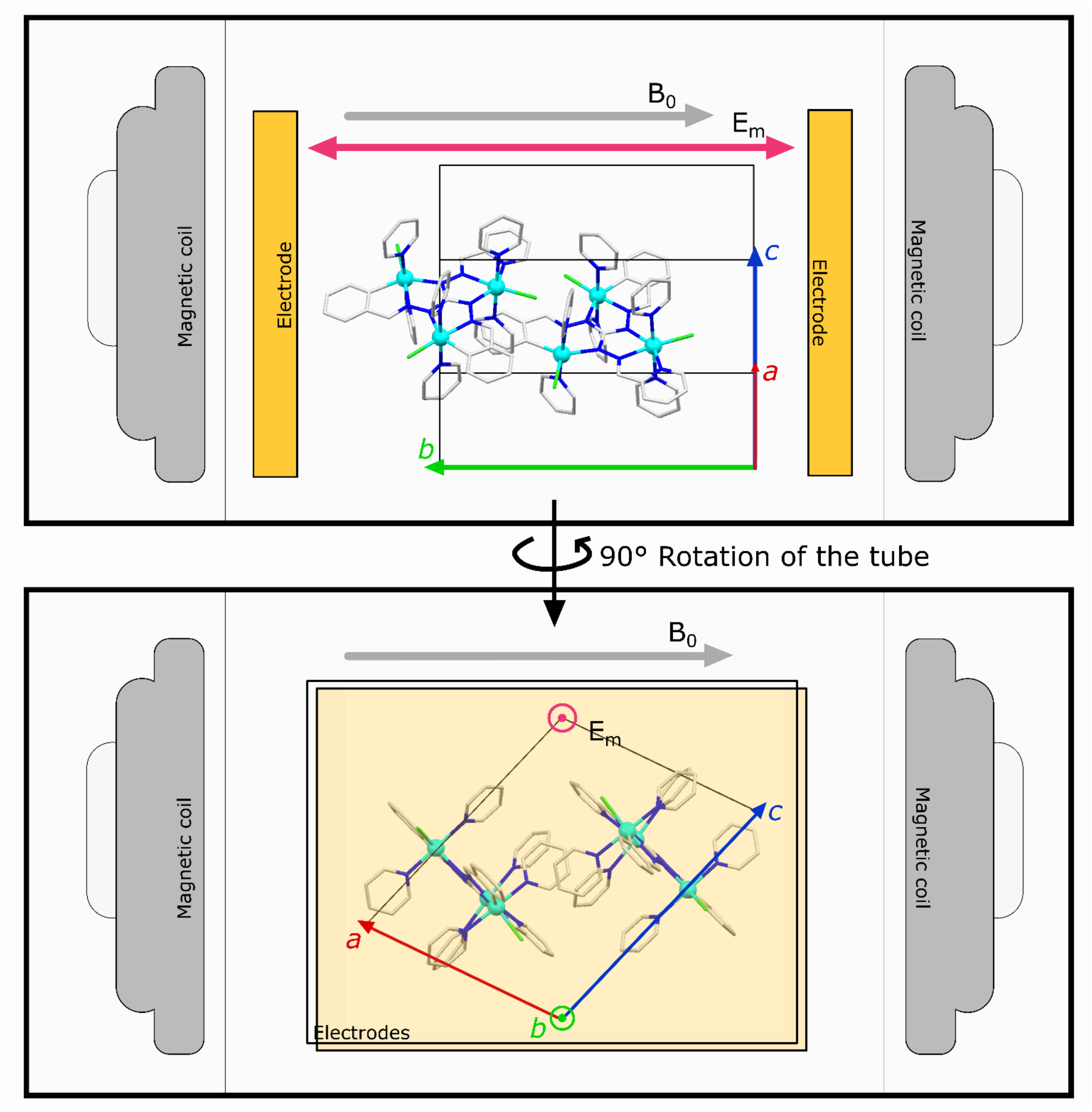

Figure S15. Schematic view of the EFM-ESR set-up and the orientation of the E_m and B_o field. The two electrodes mounted on the sample holder generate a modulated E_m field along the crystallographic $\vec{b}$ axis and thus in the Co_3 plane. The sample holder can be rotated vertically so that the B_o field can span from being parallel to perpendicular to the E_m field. However, only the first situation probes a relevant orientation.

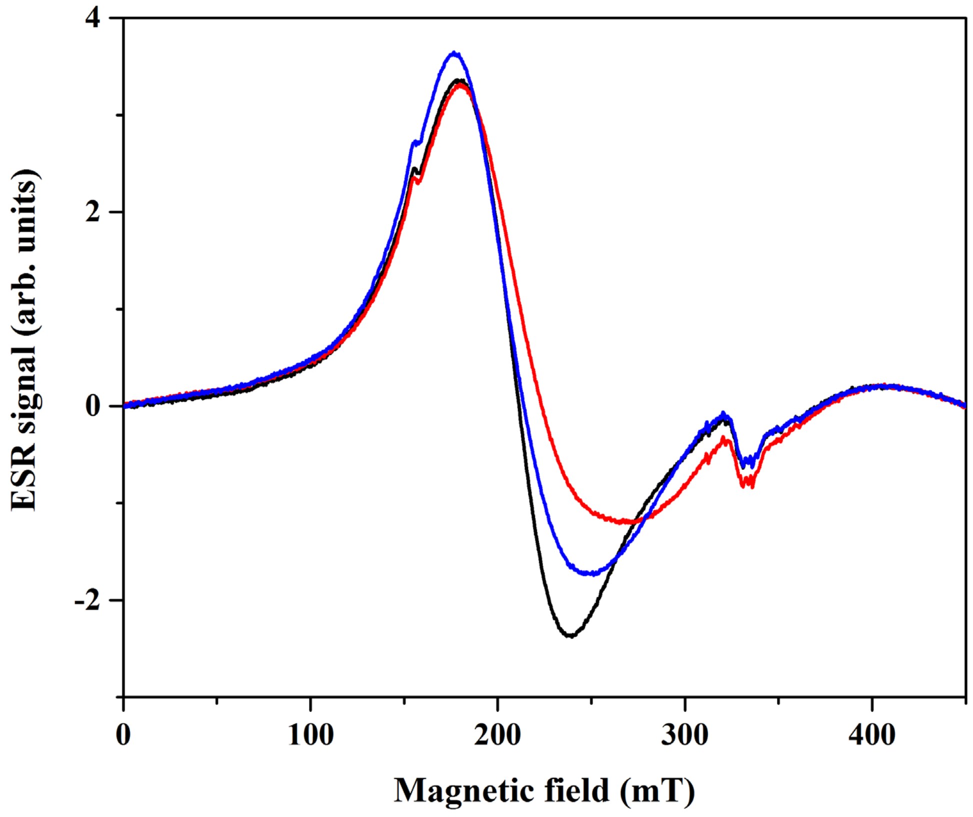

Figure S16. X-Band CW-ESR spectra of a single crystal of **Co3P** acquired at different orientations of the $\vec{b}$ axis in the *ab* plane: $\vec{b}$ parallel to the direction of B_0 (black line), $\vec{b}$ at an angle of 10° with respect to B_0 (blue line) and $\vec{b}$ at an angle of 20° with respect to B_0 (red line).

Supplementary References

[1] D. Plaul, M. Böhme, S. Ostrovsky, Z. Tomkowicz, H. Görls, W. Haase, W. Plass, *Inorg. Chem.* **2017**, *57*, 106.
[2] N. F. Chilton, R. P. Anderson, L. D. Turner, A. Soncini, K. S. Murray, *J. Comput. Chem.* **2013**, *34*, 1164.
[3] S. Stoll, A. Schweiger, *Journal of Magnetic Resonance* **2006**, *178*, 42.
[4] M. Fittipaldi, A. Cini, G. Annino, A. Vindigni, A. Caneschi, R. Sessoli, *Nat. Mater.* **2019**, *18*, 329.
[5] *TURBOMOLE V7.2 2017, a development of University of Karlsruhe and Forschungszentrum Karlsruhe GmbH, 1989 - 2007, TURBOMOLE GmbH, since 2007; available from http://www.turbomole.com.*
[6] a) J. L. Whitten, *J. Chem. Phys.* **1973**, *58*, 4496; b) C. van Alsenoy, *J. Comput. Chem.* **1988**, *9*, 620; c) E. J. Baerends, D. E. Ellis, P. Ros, *Chem. Phys.* **1973**, *2*, 41; d) B. I. Dunlap, Connolly, J. W. D., J. R. Sabin, *J. Chem. Phys.* **1979**, *71*, 3396.
[7] A. D. Becke, *Phys. Rev. A: At., Mol., Opt. Phys.* **1988**, *38*, 3098.
[8] J. P. Perdew, *Phys. Rev. B* **1986**, *33*, 8822.
[9] F. Weigend, R. Ahlrichs, *Phys. Chem. Chem. Phys.* **2005**, *7*, 3297.
[10] a) A. D. Becke, *J. Chem. Phys.* **1993**, *98*, 5648; b) C. Lee, W. Yang, R. G. Parr, *Phys. Rev. B* **1988**, *37*, 785.
[11] a) K. Yamaguchi, T. Tsunekawa, Y. Toyoda, T. Fueno, *Chem. Phys. Lett.* **1988**, *143*, 371; b) T. Soda, Y. Kitagawa, T. Onishi, Y. Takano, Y. Shigeta, H. Nagao, Y. Yoshioka, K. Yamaguchi, *Chem. Phys. Lett.* **2000**, *319*, 223.
[12] I. Fdez. Galván, M. Vacher, A. Alavi, C. Angeli, F. Aquilante, J. Autschbach, J. J. Bao, S. I. Bokarev, N. A. Bogdanov, R. K. Carlson et al., *J. Chem. Theory Comput.* **2019**, *15*, 5925.
[13] K. Andersson, B. O. Roos, *Chem. Phys. Lett.* **1992**, *191*, 507.
[14] a) L. F. Chibotaru, L. Ungur, C. Aronica, H. Elmoll, G. Pilet, D. Luneau, *J. Am. Chem. Soc.* **2008**, *130*, 12445; b) L. F. Chibotaru, L. Ungur, A. Soncini, *Angew. Chem. Int. Ed.* **2008**, *120*, 4194; c) L. Ungur, Van den Heuvel, Willem, L. F. Chibotaru, *New J. Chem.* **2009**, *33*, 1224.
[15] M. E. Lines, *J. Chem. Phys.* **1971**, *55*, 2977.
[16] H. Zabrodsky, S. Peleg, D. Avnir, *IEEE Trans. Pattern Anal. Mach. Intell.* **1995**, *17*, 1154.
[17] H. Zabrodsky, S. Peleg, D. Avnir, *J. Am. Chem. Soc.* **1993**, *115*, 8278.
[18] M. Pinsky, D. Avnir, *Inorg. Chem.* **1998**, *37*, 5575.
[19] O. Kahn, *VCH Publishers, Inc.(USA), 1993* **1993**, 393.
[20] a) K W H Stevens, *Proc. Phys. Soc.* **1952**, *65*, 209; b) C. Rudowicz, *J. Phys. C: Solid State Phys.* **1985**, *18*, 3837; c) C. Rudowicz, *J. Phys. C: Solid State Phys.* **1985**, *18*, 1415.
